# PRRSV Nsp9 hijacks BNIP3-mediated autophagy to provide membrane platforms for viral replication complexes

**DOI:** 10.1128/jvi.01314-26

**Published:** 2026-09-24

**Authors:** Heyou Yi, Shaojun Wang, Guijie Guo

**Affiliations:** 1 Key Laboratory of Animal Pathogen Infection and Immunology of Fujian Province, College of Animal Sciences, Fujian Agriculture and Forestry University 12449, Fuzhou, China; 2 Key Laboratory of Fujian-Taiwan Animal Pathogen Biology, College of Animal Sciences, Fujian Agriculture and Forestry University 12449, Fuzhou, China; 3 Joint Laboratory of Animal Pathogen Prevention and Control of Fujian-Nepal, College of Animal Sciences, Fujian Agriculture and Forestry University 12449, Fuzhou, China; 4 Guangdong Provincial Key Laboratory of Zoonosis Prevention and Control, College of Veterinary Medicine, South China Agricultural University 12526, Guangzhou, China; Loyola University Chicago - Health Sciences Campus, Maywood, Illinois, USA

**Keywords:** PRRSV, Nsp9, BNIP3, autophagy, membrane platform, viral replication

## Abstract

Porcine reproductive and respiratory syndrome virus (PRRSV) is a major pathogen threatening the global swine industry. The viral nonstructural protein 9 (Nsp9) harbors RNA-dependent RNA polymerase activity and acts as a core component of the viral replication complex, yet its interplay with host factors remains incompletely understood. In this study, we identified the mitochondrial protein BCL2 interacting protein 3 (BNIP3) as a specific binding partner of Nsp9. This interaction is mediated by the transmembrane (TM) domain of BNIP3 and the N-terminal 1–449 region of Nsp9. Functional assays demonstrated that BNIP3 facilitates PRRSV replication via its TM domain and LC3-interacting region (LIR). Mechanistically, BNIP3 recruits Nsp9 to mitochondria, where Nsp9 blocks autophagic flux and triggers incomplete mitophagy. This process leads to the accumulation of BNIP3-associated autophagic membranes, which serve as platforms for viral replication complex assembly. Moreover, simultaneous knockdown of BNIP3 and ATG7 results in further suppression of viral replication compared to knockdown of either gene alone. Rescue experiments showed that BNIP3 overexpression partially restored viral replication in ATG7-deficient cells, while ATG7 overexpression failed to rescue the replication defect caused by BNIP3 knockdown, indicating that BNIP3 possesses ATG7-independent platform functions. Collectively, our findings reveal that PRRSV hijacks BNIP3 to reprogram autophagy and generate membrane platforms for replication, which may serve as a promising target for antiviral development.

**IMPORTANCE:**

Porcine reproductive and respiratory syndrome virus (PRRSV) is one of the most economically significant viral pathogens affecting the global swine industry, yet the specific host factors that support viral replication remain incompletely defined. In this study, we identify BNIP3, a known mitochondrial protein, as a previously unrecognized interactor of the PRRSV RNA-dependent RNA polymerase Nsp9. We demonstrate that PRRSV Nsp9 exploits BNIP3 to block autophagic flux and create a specialized membrane microenvironment that serves as a membrane platform for viral replication complex assembly. Our findings reveal a mechanism by which PRRSV, a positive-sense RNA virus, utilizes a mitophagy receptor to generate replication-permissive membrane platforms, which advances our understanding of how viruses manipulate autophagy. The identification of BNIP3 as a critical host factor that promotes viral replication and the elucidation of its membrane platform function open new avenues for understanding PRRSV replication and may inform the development of novel antiviral strategies.

## INTRODUCTION

Porcine reproductive and respiratory syndrome (PRRS) is one of the most economically devastating diseases threatening the global swine industry, causing severe reproductive failure in sows and fatal respiratory distress in neonatal piglets (1–3). The causative agent, PRRS virus (PRRSV), is a positive-sense single-stranded RNA virus belonging to the family *Arteriviridae* (4, 5). Despite decades of vaccination and biosecurity measures, PRRSV remains poorly controlled, largely due to its high genetic variability and sophisticated immune evasion strategies (6–9). The viral non-structural protein 9 (Nsp9) possesses RNA-dependent RNA polymerase (RdRp) activity and is a core component of the viral replication complex, making it essential for viral RNA synthesis (10–12). Recent studies have implicated PRRSV Nsp9 in autophagosome formation, suggesting a link between this viral protein and the autophagic machinery (13, 14). However, the host factors that interact with Nsp9 to facilitate replication complex assembly are not fully understood.

BCL2 interacting protein 3 (BNIP3) is a pro-apoptotic member of the Bcl-2 family and a well-known mitophagy receptor (15, 16). Under hypoxic or stress conditions, BNIP3 localizes to the outer mitochondrial membrane via its C-terminal transmembrane (TM) domain and recruits LC3 through its LIR (LC3-interacting region) motif, thereby targeting damaged mitochondria for autophagic degradation (17–19). Several studies have shown that BNIP3 also participates in the replication of several viruses, including Chikungunya virus, Enterovirus 71, Southern rice black-streaked dwarf virus, Singapore grouper iridovirus, and Bovine papillomavirus (20–24). However, whether PRRSV engages BNIP3 to support its replication and the underlying molecular mechanism remain largely unexplored.

Autophagy is a catabolic process that delivers cytoplasmic components to lysosomes for degradation and recycling (25, 26). Accumulating evidence indicates that many viruses, including PRRSV, manipulate the autophagic machinery to promote their own replication (27, 28). PRRSV infection induces incomplete autophagy, characterized by accumulation of autophagosomes without efficient degradation, and this process facilitates viral replication (29, 30). Nevertheless, the precise molecular mechanism by which PRRSV exploits autophagic membranes for replication complex formation has not been established. In particular, it is unknown whether PRRSV Nsp9 interacts with a specific autophagy receptor to gain access to membrane platforms.

In this study, we identified BNIP3 as a novel host interactor of PRRSV Nsp9. We demonstrate that Nsp9 specifically binds to the TM domain of BNIP3 through its N-terminal 1–449 amino acid region, thereby recruiting Nsp9 to the mitochondrial membrane. BNIP3 directly binds LC3 via its LIR motif, a key step in autophagy regulation, and the Nsp9-BNIP3 axis is essential for the accumulation of autophagic membranes induced by PRRSV. Importantly, we show that BNIP3-associated autophagic membranes serve as platforms for the assembly of viral replication complexes, as evidenced by colocalization of viral dsRNA, mitochondria, and BNIP3. Disruption of the Nsp9-BNIP3 interaction disrupts membrane association of replication complexes and severely impairs viral replication. Furthermore, BNIP3 acts cooperatively with ATG7 to sustain autophagic membranes utilized during infection, and the ability of BNIP3 to promote viral replication strictly depends on its LIR motif. Together, our findings reveal a previously unrecognized mechanism by which PRRSV Nsp9 hijacks BNIP3-mediated autophagy to acquire membrane platforms for replication complex assembly, highlighting BNIP3 as a potential target for antiviral intervention.

## RESULTS

### BNIP3 specifically interacts with PRRSV Nsp9

**Fig 1:**
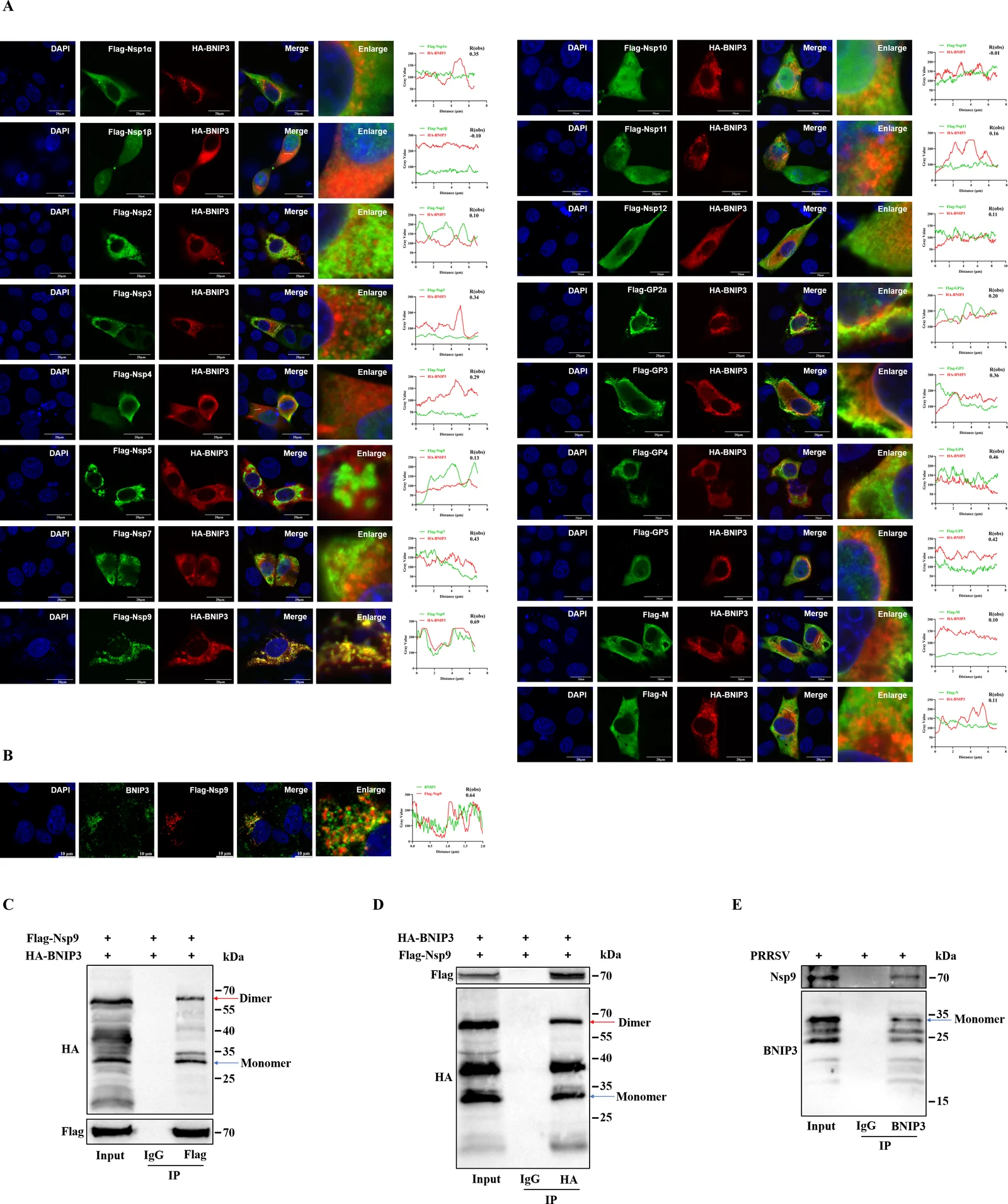
BNIP3 specifically interacts with PRRSV Nsp9. (**A**) Marc-145 cells were co-transfected with HA-BNIP3 and individual Flag-tagged PRRSV proteins for 24 h. Cells were stained with rabbit anti-HA and mouse anti-Flag antibodies, and nuclei were counterstained with DAPI. Confocal microscopy was used to acquire images. Fluorescence intensity profiles and Pearson’s correlation coefficient (Robs) were analyzed using ImageJ 1.8.0. Scale bar, 20 μm. (**B**) Marc-145 cells were transfected with Flag-Nsp9 for 24 h. Endogenous BNIP3 and Flag-Nsp9 were detected using mouse anti-BNIP3 and rabbit anti-Flag antibodies, respectively, followed by DAPI nuclear staining. Colocalization and fluorescence intensity were quantified with ImageJ 1.8.0. Scale bar, 20 μm. (**C and D**) HEK-293T cells were co-transfected with HA-BNIP3 and Flag-Nsp9 for 24 h. Cell lysates were subjected to co-immunoprecipitation (Co-IP) using mouse anti-Flag (**C**) or mouse anti-HA antibody (**D**). Precipitated proteins were analyzed by Western blotting with rabbit anti-HA and rabbit anti-Flag antibodies. Normal mouse IgG was used as a negative control (NC). (**E**) Marc-145 cells were infected with PRRSV for 8 h. Cell lysates were immunoprecipitated (IP) with mouse anti-BNIP3 antibody or control IgG, followed by immunoblotting (IB) with alpaca anti-Nsp9 and rabbit anti-BNIP3 antibodies. Input lysates were analyzed as a loading control.

To explore whether PRRSV utilizes the host protein BNIP3 during infection, we screened for BNIP3-interacting viral proteins by confocal microscopy. Marc-145 cells were co-transfected with BNIP3 and individual PRRSV proteins, including Nsp1α, Nsp1β, Nsp2, Nsp3, Nsp4, Nsp5, Nsp7, Nsp9, Nsp10, Nsp11, Nsp12, GP2a, GP3, GP4, GP5, M, and N. Fluorescence intensity profiling and Pearson’s correlation coefficient (Robs) analysis revealed that Nsp9, but not other viral proteins, showed significant colocalization with BNIP3 (Robs >0.50) (Fig. 1A). Consistent with these results, endogenous BNIP3 also colocalized with exogenous Nsp9 (Robs >0.50) (Fig. 1B).

Co-immunoprecipitation (Co-IP) assays were further performed to verify this interaction. Exogenously expressed BNIP3 specifically bound to Nsp9 (Fig. 1C and D). Moreover, endogenous Nsp9 was specifically co-precipitated with endogenous BNIP3 in PRRSV-infected cells, whereas no signal was detected in the IgG control or in mock-infected cells (Fig. 1E). A similar result was obtained using the heterologous NADC30-like strain (Fig. S1A), indicating that the BNIP3-Nsp9 interaction is conserved across different PRRSV strains. Collectively, these data demonstrate that BNIP3 specifically interacts with PRRSV Nsp9 in host cells.

### The TM domain of BNIP3 and the N-terminal 1–449 aa region of Nsp9 are critical for their interaction

**Fig 2:**
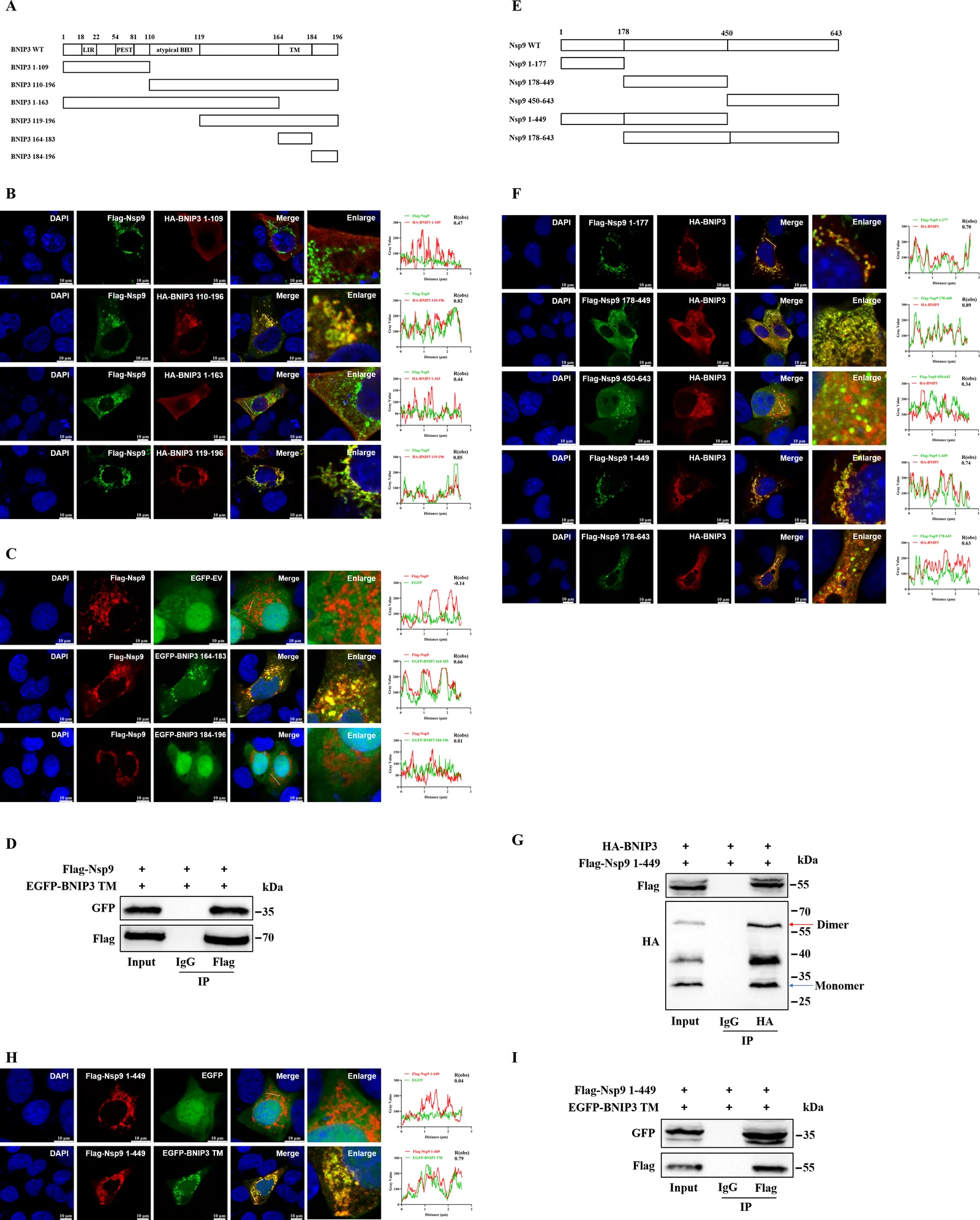
The TM domain of BNIP3 and the 1–449 aa region of Nsp9 mediate their specific interaction. (**A**) Schematic representation of full-length BNIP3 and its truncated mutants: 1–109 aa, 110–196 aa, 1–163 aa, 119–196 aa, 164–183 aa (TM domain), and 184–196 aa. (**B and C**) Marc-145 cells were co-transfected with Flag-Nsp9 and HA-tagged BNIP3 truncates (**B**) or EGFP-BNIP3 fragments (**C**). Cells were fixed at 24 h post-transfection and stained with corresponding antibodies; EGFP signals were detected directly. Nuclei were labeled with DAPI. Scale bar, 10 μm. (**D**) HEK-293T cells were co-transfected with Flag-Nsp9 and EGFP-BNIP3 TM (164–183 aa) for 24 h. Cell lysates were used for co-immunoprecipitation with mouse anti-Flag antibody. Precipitated proteins were detected by Western blotting using rabbit anti-Flag and rabbit anti-GFP antibodies. (**E**) Schematic diagram of Nsp9 truncation mutants, including 1–177 aa, 178–449 aa, 450–643 aa, 1–449 aa, and 178–643 aa. (**F**) Marc-145 cells were co-transfected with HA-BNIP3 and individual Flag-tagged Nsp9 truncates for 24 h. Cells were stained with rabbit anti-HA and mouse anti-Flag antibodies, and nuclei were counterstained with DAPI. Scale bar, 10 μm. (**G**) HEK-293T cells were co-transfected with HA-BNIP3 and Flag-Nsp9 1–449 aa truncated fragment for 24 h. Cell lysates were subjected to Co-IP using mouse anti-Flag antibody, and bound proteins were analyzed by Western blotting with rabbit anti-HA and rabbit anti-Flag antibodies. (**H**) Marc-145 cells were co-transfected with EGFP-BNIP3 TM and Nsp9 1–449 aa truncated fragment for 24 h. EGFP signals were visualized directly, and Nsp9 was detected using mouse anti-Flag antibody. Nuclei were counterstained with DAPI. Scale bar, 10 μm. (**I**) HEK-293T cells were co-transfected with EGFP-fused BNIP3 TM domain and Flag-Nsp9 1–449 aa for 24 h. Cell lysates were immunoprecipitated with mouse anti-Flag antibody, followed by Western blotting with rabbit anti-GFP and rabbit anti-Flag antibodies. For all colocalization assays (**B, C, F, and H**), fluorescence intensity profiles and Pearson’s correlation coefficients (Robs) were quantified using ImageJ 1.8.0.

To identify the key domains responsible for the BNIP3-Nsp9 interaction, a series of BNIP3 truncation mutants were constructed, including 1–109 aa, 110–196 aa, 1–163 aa, 119–196 aa, 164–183 aa (transmembrane [TM] domain), and 184–196 aa (Fig. 2A). Each mutant was co-transfected with Flag-Nsp9, and colocalization was analyzed and quantified using fluorescence intensity profiles and Pearson’s correlation coefficient (Robs). BNIP3 mutants containing the TM domain (110–196 aa and 119–196 aa) exhibited strong colocalization with Nsp9 (Robs >0.80), while mutants lacking the TM domain (1–109 aa, 1–163 aa, and 184–196 aa) displayed markedly reduced colocalization (Robs <0.50) (Fig. 2B and C). Notably, the TM domain alone (164–183 aa) was sufficient to colocalize with Nsp9 (Robs >0.60) (Fig. 2C). Co-immunoprecipitation assays further validated that the BNIP3 TM domain efficiently binds to Nsp9 (Fig. 2D). Collectively, these data indicate that the TM domain is the essential region of BNIP3 for Nsp9 binding.

To further map the BNIP3-interacting region of Nsp9, multiple Nsp9 truncation mutants (1–177 aa, 178–449 aa, 450–643 aa, 1–449 aa, and 178–643 aa) were generated (Fig. 2E). Colocalization analysis demonstrated that Nsp9 mutants covering the N-terminal region (1–177 aa and 178–449 aa) exhibited strong colocalization with BNIP3 (Robs >0.60), whereas the C-terminal fragment (450–643 aa) showed negligible colocalization (Robs <0.50) (Fig. 2F). Consistently, Co-IP experiments confirmed that the Nsp9 1–449 aa region is responsible for BNIP3 binding (Fig. 2G). Furthermore, confocal microscopy and Co-IP assays using only the BNIP3 TM domain and Nsp9 1–449 aa fragment verified their direct interaction (Fig. 2H and I). Taken together, these results demonstrate that BNIP3 recognizes Nsp9 via its TM domain, while Nsp9 interacts with BNIP3 through its N-terminal 1–449 aa region, thereby mediating their specific binding.

### BNIP3 positively regulates PRRSV replication in a TM domain-dependent manner

**Fig 3:**
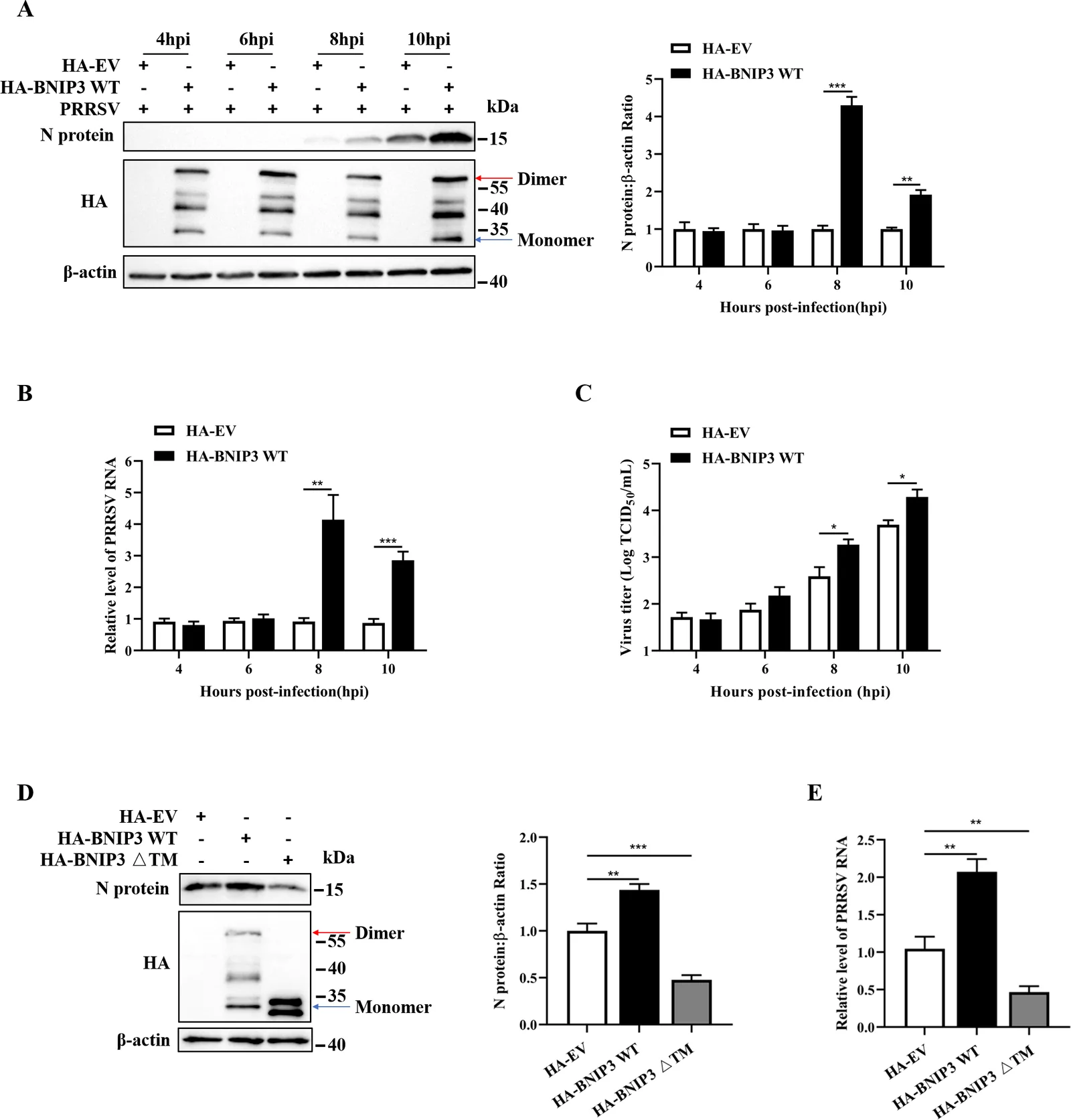
BNIP3 promotes PRRSV replication via its TM domain. (**A and B**) Marc-145 cells were transfected with HA-BNIP3 or HA-empty vector (HA-EV) for 24 h, followed by PRRSV infection for 4, 6, 8, and 10 h. Viral N protein expression was analyzed by Western blotting (**A**), viral RNA levels were quantified by RT-qPCR (**B**), and viral titers in culture supernatants were determined by TCID_50_ assay (**C**). (**D and E**) Marc-145 cells were transfected with HA-BNIP3 WT, HA-BNIP3 ΔTM, or HA-EV for 24 h prior to 8 h of PRRSV infection. Viral N protein and viral RNA were detected by Western blotting (**D**) and RT-qPCR (**E**), respectively. Data are presented as mean ± SD. *P < 0.05; **P < 0.01; ***P < 0.001.

Time points (4–10 hours post-infection [hpi]) were chosen to capture the early-to-mid phase of PRRSV replication, as later time points (12–24 hpi) were associated with extensive cytopathic effects (CPE) under the multiplicity of infection (MOI) conditions used (MOI = 10). At 4–6 hpi, viral RNA and protein levels were relatively low, so 8 and 10 hpi were selected as the primary time points for analysis. To investigate the effect of BNIP3 on PRRSV replication, Marc-145 cells were transfected to overexpress wild-type BNIP3 (BNIP3 WT) or TM domain-deleted BNIP3 mutant (BNIP3 ΔTM). Western blotting and RT-qPCR results showed that BNIP3 WT significantly enhanced viral N protein expression and RNA levels at 8 and 10 hpi (Fig. 3A and B), and TCID_50_ assay further confirmed that BNIP3 WT increased infectious virus production (Fig. 3C). In contrast, BNIP3 ΔTM overexpression failed to facilitate viral replication and markedly reduced viral N protein and RNA levels (Fig. 3D and E). These findings indicate that BNIP3 promotes PRRSV replication strictly dependent on its TM domain.

**Fig 4:**
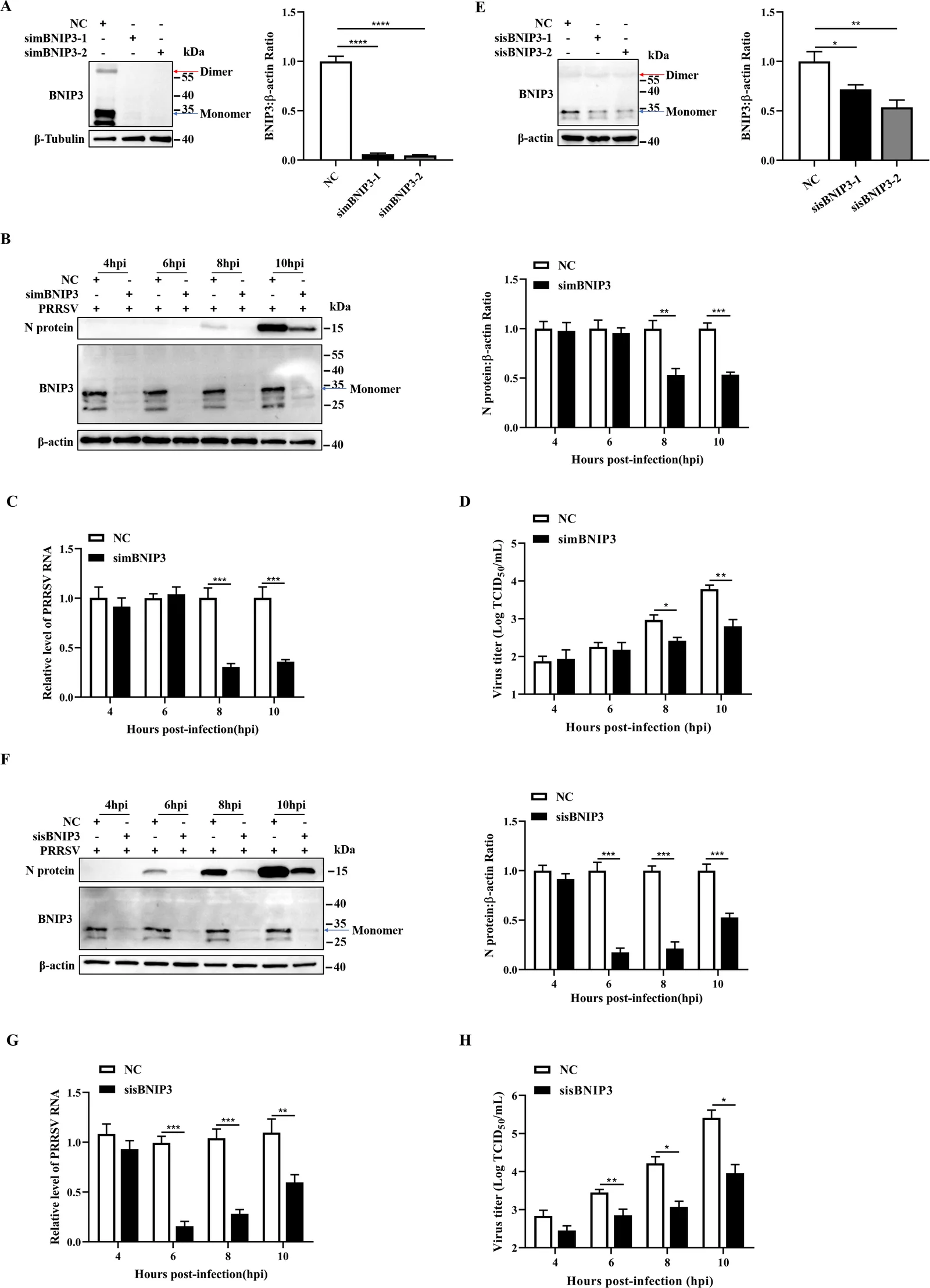
BNIP3 knockdown suppresses PRRSV replication. (**A and E**) Marc-145 cells transfected with simBNIP3 (**A**) and primary PAMs transfected with sisBNIP3 (**E**), along with NC siRNA, were cultured for 48 h. BNIP3 knockdown efficiency in both cell types was verified by Western blotting. (**B–D and F–H**) After 48 h of siRNA transfection, both cell types were infected with PRRSV for 4, 6, 8, and 10 h. Viral N protein, RNA levels, and viral titers were assessed in simBNIP3-treated Marc-145 cells (**B–D**) and sisBNIP3-treated PAMs (**F–H**) via Western blotting, RT-qPCR, and TCID_50_ assay, respectively. Data are presented as mean ± SD. *P < 0.05; **P < 0.01; ***P < 0.001; ****P < 0.0001.

Efficient BNIP3-specific siRNAs were selected for Marc-145 cells (simBNIP3-2) and primary porcine alveolar macrophages (PAMs) (sisBNIP3-2) from two candidates each (Fig. 4A and E). Knockdown of endogenous BNIP3 with these siRNAs markedly reduced viral N protein, RNA levels, and infectious virus titers at 8 and 10 h post-infection in both Marc-145 cells (Fig. 4B through D) and PAMs (Fig. 4F through H). These results confirm that BNIP3 positively regulates PRRSV replication in both Marc-145 cells and primary PAMs, the natural target cells of PRRSV.

To further determine whether BNIP3 promotes replication of heterologous PRRSV strains, we overexpressed or knocked down BNIP3 in Marc-145 cells prior to infection with the NADC30-like strain. Since the NADC30-like strain replicates with slower kinetics and produces lower viral titers compared to the XHGD strain used in the preceding experiments, we analyzed samples at 12, 24, 36, and 48 h post-infection instead of the earlier time points used for XHGD. Overexpression of HA-BNIP3 significantly enhanced NADC30-like N protein expression and viral titers at 24, 36, and 48 hpi, whereas BNIP3 knockdown markedly reduced both N protein levels and viral titers at the same time points (Fig. S2 and S3). No significant differences were observed at 12 hpi. These results confirm that BNIP3 promotes PRRSV replication across different viral strains.

### Nsp9 induces incomplete mitophagy in a BNIP3-dependent manner

During autophagic flux, autophagosomes fuse with lysosomes to degrade their cargo. To examine whether Nsp9 affects this process, we employed a tandem fluorescent reporter, LC3-GFP-mCherry. Upon delivery to the acidic lysosomal compartment, the GFP signal is quenched while mCherry remains detectable. Thus, red-only puncta represent functional autolysosomes with completed autophagic flux, whereas yellow puncta, resulting from colocalized GFP and mCherry, indicate autophagosomes that have not fused with lysosomes or have impaired acidification, a state known as incomplete autophagy.

**Fig 5:**
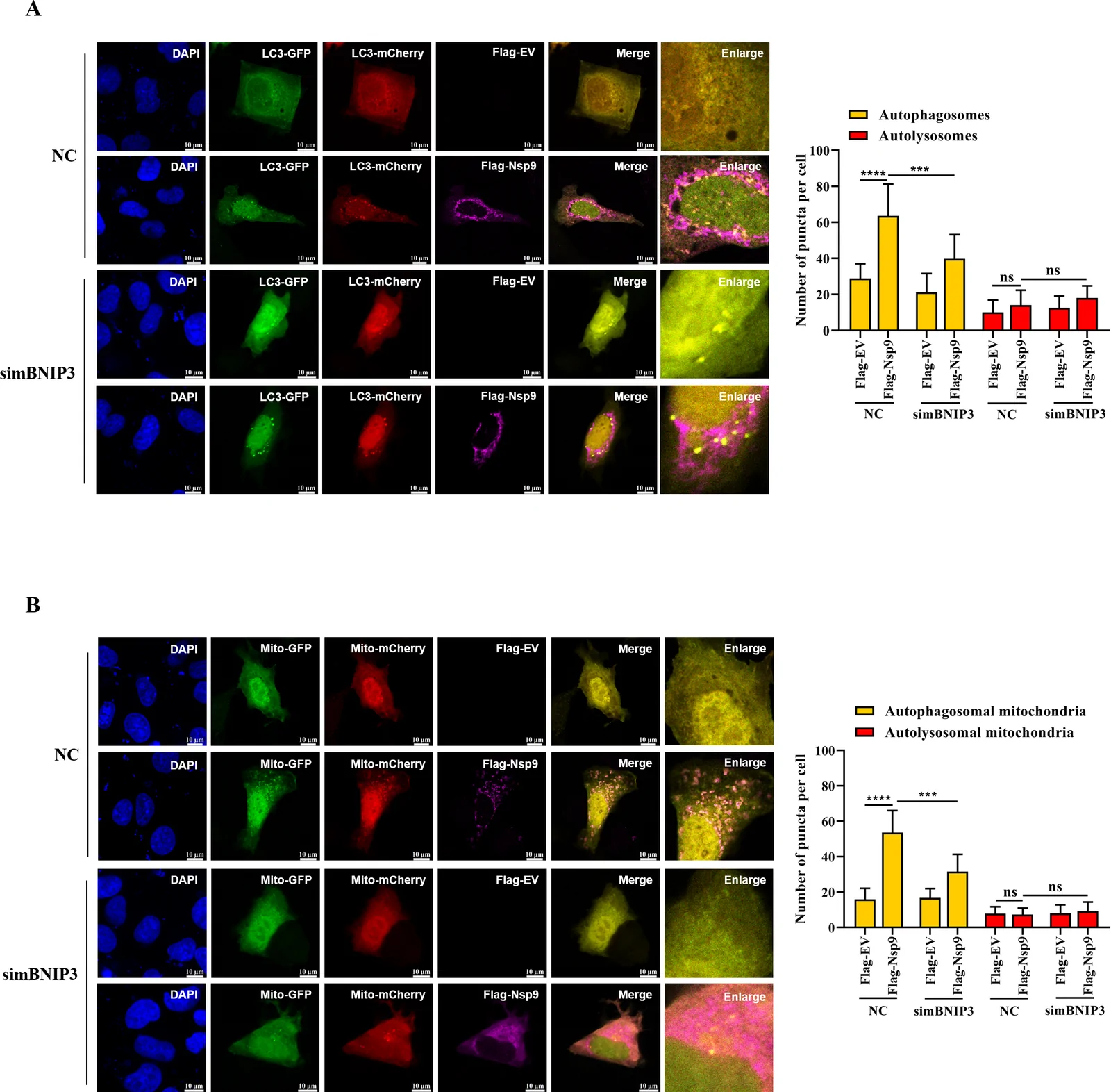
Nsp9 induces incomplete autophagy via BNIP3. Marc-145 cells were transfected with control siRNA (NC) or simBNIP3 for 48 h and then co-transfected with Flag-EV or Flag-Nsp9, along with either LC3-GFP-mCherry (**A**) for autophagic flux detection or Mito-GFP-mCherry (**B**) for mitophagic flux detection. After 24 h, cells were observed under confocal microscopy. Yellow puncta (GFP^+^mCherry^+^) represent autophagosomes or undegraded mitochondria, while red-only puncta (GFP⁻mCherry^+^) indicate autolysosomes or lysosome-delivered mitochondria. At least 20 cells per group from three independent experiments were quantified. Statistical significance was determined by Student’s *t*-test for the indicated comparisons. Scale bar, 10 μm. Data are presented as mean ± SD. ***P < 0.001; ****P < 0.0001; ns, not significant..

Marc-145 cells were transfected with control siRNA (NC) or simBNIP3 for 48 h, followed by co-transfection with LC3-GFP-mCherry and either Flag-empty vector (Flag-EV) or Flag-Nsp9 for an additional 24 h. Confocal microscopy analysis revealed that control cells displayed a basal level of autophagic flux (Fig. 5A, top row). Nsp9-expressing cells showed a marked accumulation of yellow puncta, whereas red-only puncta remained comparable to the control (Fig. 5A, second row). If autophagosome formation were simply increased while flux remained intact, a corresponding increase in red-only puncta (autolysosomes) would be expected. The fact that red-only puncta remained unchanged indicates that the newly formed autophagosomes are not maturing into autolysosomes, consistent with a block in autophagic flux rather than merely enhanced autophagosome biogenesis, confirming that Nsp9 blocks autophagic flux. BNIP3-knockdown cells exhibited a puncta pattern similar to that of control cells, indicating that BNIP3 knockdown alone did not obviously affect basal autophagic flux (Fig. 5A, third row). Notably, Nsp9-expressing BNIP3-knockdown cells showed significantly fewer yellow puncta than Nsp9-expressing cells although yellow puncta remained slightly elevated compared to BNIP3-knockdown cells (Fig. 5A, bottom row). These results demonstrate that Nsp9 blocks autophagic flux in a BNIP3-dependent manner, leading to accumulation of autophagosomes and induction of incomplete autophagy.

To determine whether Nsp9 also interferes with mitophagic flux, we utilized a mitochondrially targeted tandem fluorescent reporter, Mito-GFP-mCherry. Consistent with the LC3-GFP-mCherry results, BNIP3 knockdown significantly reduced Nsp9-induced accumulation of yellow puncta (Fig. 5B, bottom row). These data demonstrate that Nsp9 requires BNIP3 to impair the delivery of mitochondria to lysosomes, a state defined as incomplete mitophagy.

### BNIP3 is required for PRRSV-induced autophagic flux blockade

To investigate the role of BNIP3 in PRRSV-mediated autophagic flux inhibition, we performed autophagic flux assays using Bafilomycin A1 (BafA1). BafA1 blocks autophagosome–lysosome fusion and suppresses autophagic degradation, thereby causing LC3-II accumulation. Accordingly, the difference in LC3-II levels with or without BafA1 treatment reflects the dynamic status of autophagic flux.

**Fig 6:**
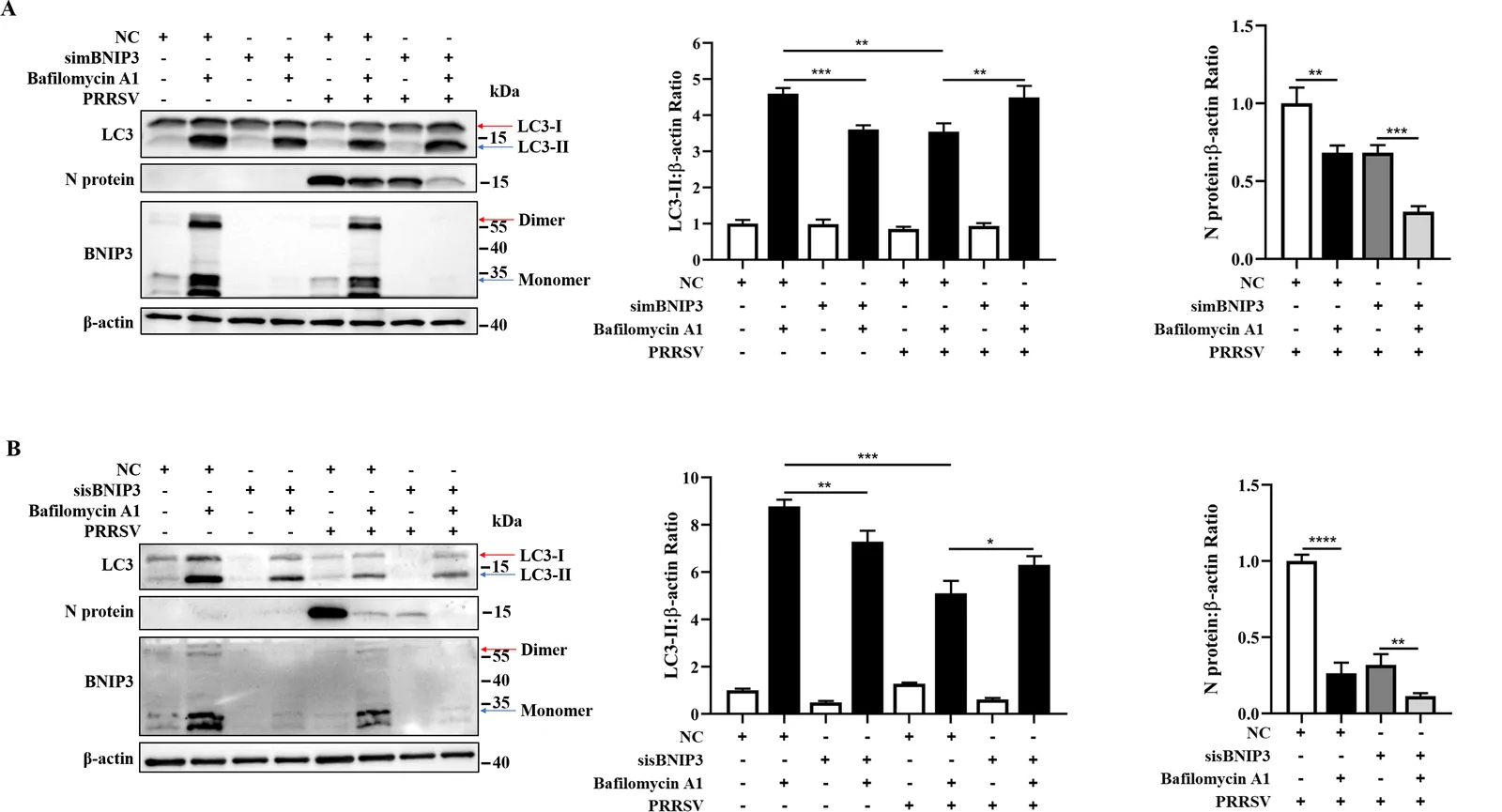
BNIP3 is required for PRRSV-induced autophagic flux blockade. Marc-145 cells (**A**) and primary porcine alveolar macrophages (PAMs) (**B**) were transfected with negative control siRNA (NC) or cell-specific BNIP3 siRNA (simBNIP3 for Marc-145 cells, sisBNIP3 for PAMs) for 48 h, followed by mock infection or PRRSV infection for 8 h. For the final 4 h before cell collection, cells were treated with 100 nM Bafilomycin A1 (BafA1) or DMSO vehicle control. The protein levels of LC3-II and viral N were determined by Western blotting. Data are presented as mean ± SD. *P < 0.05; **P < 0.01; ***P < 0.001; ****P < 0.0001.

In uninfected Marc-145 cells, BNIP3 knockdown diminished the LC3-II response to BafA1 compared to the negative control (NC) (Fig. 6A, compare lanes 4 and 2), indicating that BNIP3 contributes to basal autophagic flux. In PRRSV-infected Marc-145 cells, the NC control group showed a reduced LC3-II response to BafA1 compared to uninfected cells (Fig. 6A, compare lane 6 with lane 2). This reduced response occurs because PRRSV infection itself blocks autophagosome-lysosome fusion, thereby suppressing autophagic turnover. Under these conditions, the pool of newly forming autophagosomes available for BafA1-induced accumulation is diminished, resulting in lower LC3-II levels upon BafA1 treatment compared to uninfected cells. These results confirm that PRRSV infection inhibits autophagic flux. Upon BNIP3 knockdown, the LC3-II response to BafA1 was markedly increased, accompanied by a decrease in viral N protein expression (Fig. 6A, compare lane 8 with lane 6). These results suggest that BNIP3 knockdown relieves the virus-mediated autophagic flux blockade, partially restores flux, and thereby enables a stronger LC3-II accumulation response to BafA1.

We repeated the experiments in PAMs. Consistent with the observations in Marc-145 cells, PRRSV-infected PAMs transfected with NC siRNA displayed a reduced LC3-II response to BafA1, indicating virus-induced autophagic flux blockade (Fig. 6B, compare lane 6 with lane 2). In contrast, BNIP3 knockdown significantly enhanced the LC3-II response to BafA1 and reduced viral N protein expression (Fig. 6B, compare lane 8 with lane 6). Collectively, these data demonstrate that BNIP3 is a critical host factor required for PRRSV-induced autophagic flux blockade; the virus exploits BNIP3 to suppress autophagic flux, thus creating an incomplete autophagy environment favorable for viral replication.

To further assess whether pharmacological activation of autophagy could bypass the requirement for BNIP3, we treated cells with rapamycin, a well-established autophagy inducer, in the presence or absence of BNIP3 knockdown and PRRSV infection (Fig. S4). Rapamycin induced LC3-II accumulation across all conditions, confirming its autophagic activity. However, rapamycin treatment did not enhance PRRSV replication; instead, it significantly reduced viral N protein expression and viral titers (Fig. S4A and B). Notably, BNIP3 knockdown alone yielded a similar level of suppression, and combined treatment with rapamycin further reduced viral replication to the lowest level among all groups. These results demonstrate that pharmacological activation of autophagy cannot compensate for the loss of BNIP3, reinforcing the conclusion that BNIP3-mediated membrane platform formation is a rate-limiting step in PRRSV replication.

### BNIP3 promotes PRRSV replication in a LIR-dependent manner

**Fig 7:**
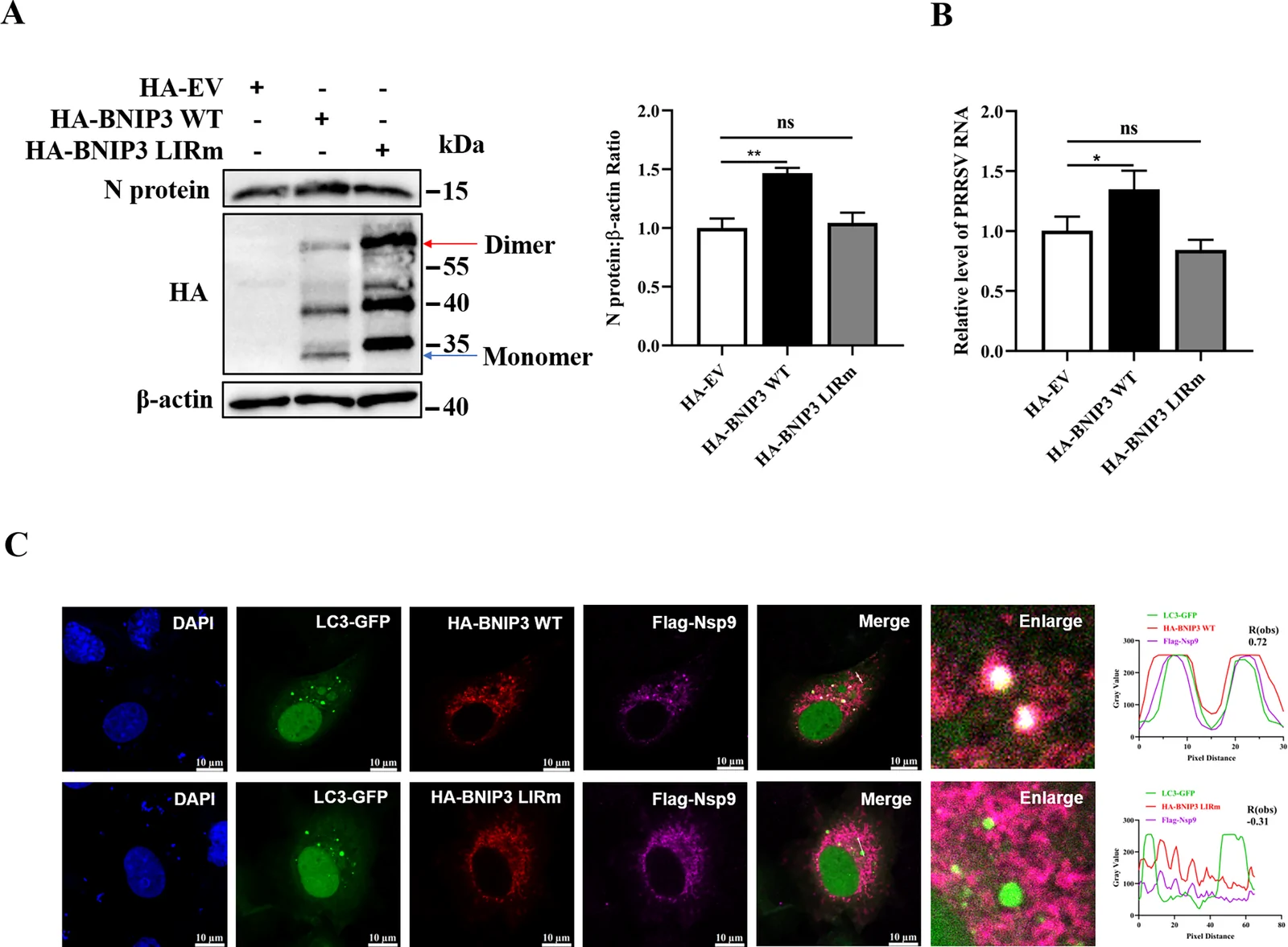
BNIP3 promotes PRRSV replication in a LIR-dependent manner. (**A and B**) Marc-145 cells were transfected with HA-empty vector (HA-EV), HA-BNIP3 WT, or HA-BNIP3 LIRm for 24 h, followed by PRRSV infection for 8 h. Cell lysates were harvested for Western blotting to detect HA-tagged proteins and viral N protein (**A**), and viral RNA levels were quantified by RT-qPCR (**B**). (**C**) Marc-145 cells were co-transfected with LC3-GFP, Flag-Nsp9, and either HA-BNIP3 WT or HA-BNIP3 LIRm for 24 h. Cells were immunostained with rabbit anti-HA and mouse anti-Flag primary antibodies, followed by fluorescent secondary antibodies. Nuclei were counterstained with DAPI. Fluorescence intensity profiles and Pearson’s correlation coefficients (Robs) were analyzed using ImageJ 1.8.0. Scale bar, 10 μm. Data are presented as mean ± SD. *P < 0.05; **P < 0.01; ns, not significant.

To determine whether BNIP3 facilitates viral replication through binding to LC3, Marc-145 cells were transfected with HA-empty vector (HA-EV), HA-BNIP3 wild-type (WT), or HA-BNIP3 LIR motif (LIRm) mutant and then infected with PRRSV. Western blotting showed that BNIP3 WT significantly increased viral N protein expression, whereas the LIRm mutant failed to promote viral protein production (Fig. 7A). RT-qPCR analysis of viral RNA yielded consistent results (Fig. 7B). These data indicate that BNIP3 promotes PRRSV replication strictly in an LIR motif-dependent manner.

To further dissect the functional role of the LIR motif, we performed colocalization analysis in cells co-expressing LC3-GFP, Flag-Nsp9, and either HA-BNIP3 WT or HA-BNIP3 LIRm. The three proteins exhibited robust colocalization in the BNIP3 WT group (Fig. 7C, top). In contrast, the BNIP3 LIRm retained colocalization with Nsp9 but completely abolished its association with LC3 (Fig. 7C, bottom). These results confirm that the BNIP3 LIR motif specifically mediates LC3 binding, while not affecting the BNIP3–Nsp9 interaction. Taken together, BNIP3 facilitates PRRSV replication through LIR-dependent LC3 engagement.

### Nsp9 is recruited to mitochondria via BNIP3 to form replication platforms

**Fig 8:**
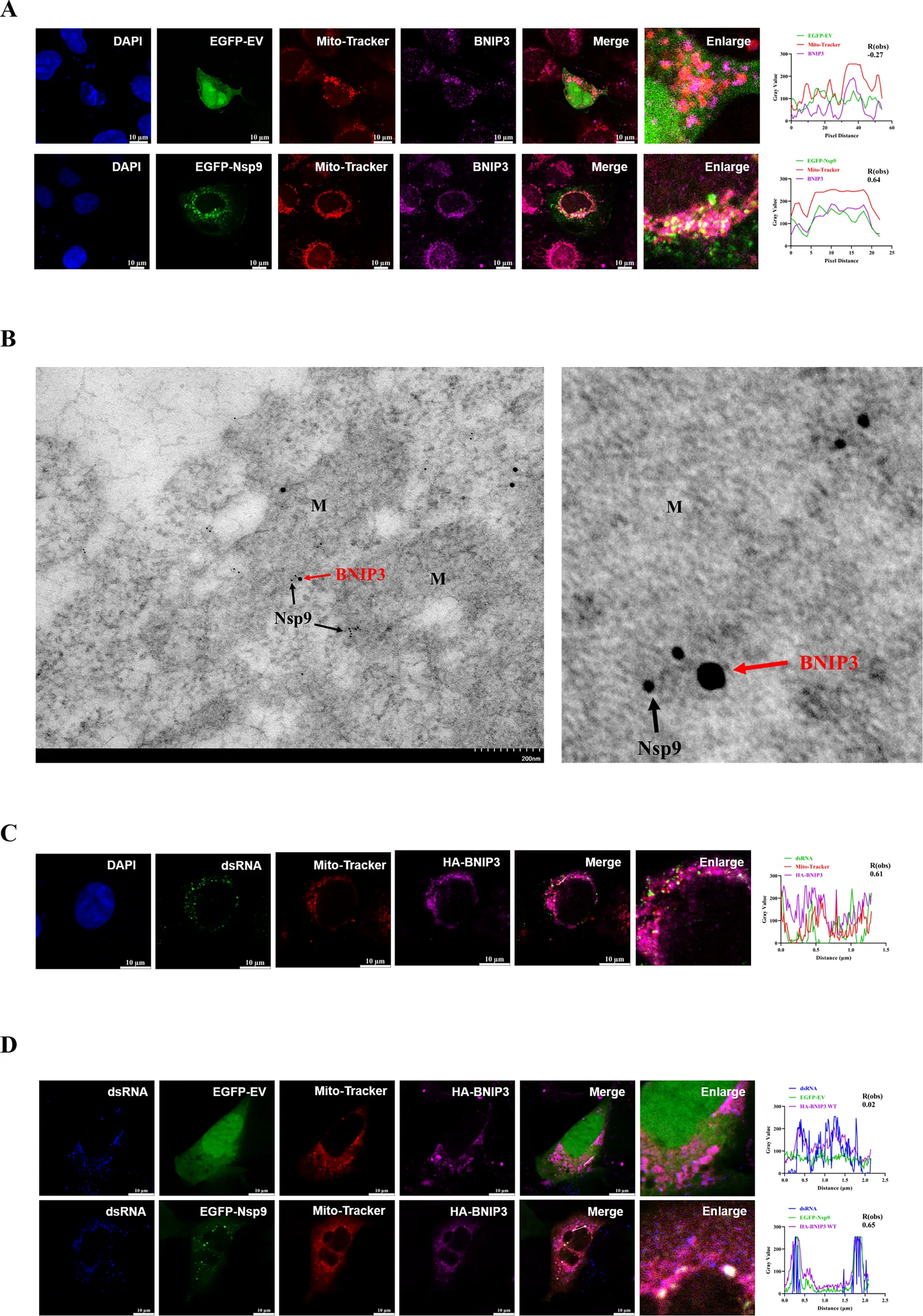
Nsp9–BNIP3 complex localizes to mitochondrial membranes. (**A**) Marc-145 cells were transfected with EGFP empty vector (EGFP-EV) or EGFP-Nsp9 for 24 h. Mitochondria were labeled with Mito-Tracker, and endogenous BNIP3 was stained with mouse anti-BNIP3 primary antibody and Alexa Fluor 647-conjugated secondary antibody. Confocal images were acquired, and fluorescence distribution and colocalization were quantified using ImageJ 1.8.0. Scale bar, 10 μm. (**B**) Marc-145 cells co-expressing Flag-Nsp9 and HA-BNIP3 were processed for immunogold labeling. Samples were dual-labeled with anti-Flag (4-nm gold particles) and anti-HA (12-nm gold particles). Electron micrographs revealed that Nsp9 (4-nm, black arrows) and BNIP3 (12-nm, red arrows) colocalized closely at the outer mitochondrial membrane. Scale bar, 200 nm. (**C**) Marc-145 cells were transfected with HA-BNIP3 for 24 h and then infected with PRRSV for 8 h. Cells were co-stained with mouse anti-dsRNA, rabbit anti-HA antibodies, and Mito-Tracker. Nuclei were counterstained with DAPI. The colocalization of viral dsRNA, mitochondria, and HA-BNIP3 was analyzed by confocal microscopy. Scale bar, 10 μm. (**D**) Marc-145 cells were co-transfected with EGFP-Nsp9 or EGFP-EV and HA-BNIP3 for 24 h and then infected with PRRSV for 8 h. Cells were stained with mouse anti-dsRNA antibody, rabbit anti-HA antibody, and Mito-Tracker. EGFP-Nsp9 fluorescence was directly visualized. Scale bar, 10 μm.

Three-color colocalization analysis demonstrated that Flag-Nsp9, Mito-Tracker-labeled mitochondria, and endogenous BNIP3 colocalized within the same subcellular regions (Fig. 8A). Immunoelectron microscopy further visualized the subcellular localization of the BNIP3–Nsp9 complex. In Marc-145 cells co-expressing Flag-Nsp9 and HA-BNIP3, 4-nm gold particles (Nsp9, black arrows) and 12-nm gold particles (BNIP3, red arrows) were predominantly distributed on the outer mitochondrial membrane and exhibited close spatial proximity, confirming the formation of nanoscale BNIP3–Nsp9 complexes at mitochondrial membranes (Fig. 8B).

Three-color confocal microscopy of PRRSV-infected cells showed that viral dsRNA (a marker of active replication complexes), mitochondria, and HA-BNIP3 colocalized, indicating that viral replication occurs on BNIP3-associated mitochondrial membranes (Fig. 8C). To further confirm that the BNIP3-associated mitochondrial membranes serve as sites for functional replication complexes, we examined the spatial relationship between Nsp9, dsRNA, BNIP3, and mitochondria. Marc-145 cells were co-transfected with EGFP-Nsp9 (or EGFP-EV as control) and HA-BNIP3 and then infected with PRRSV for 8 h. Confocal microscopy revealed that EGFP-Nsp9 colocalized with dsRNA, HA-BNIP3, and mitochondria in infected cells (Fig. 8D). In contrast, no such colocalization was observed in cells expressing EGFP-EV (Fig. 8D). These results demonstrate that Nsp9, as a core component of the viral replication complex, is present at the BNIP3-associated mitochondrial membranes where active viral RNA synthesis occurs, providing evidence that these membranes serve as platforms for functional replication complexes.

### BNIP3 and ATG7 cooperatively regulate PRRSV replication

To investigate the functional relationship between BNIP3 and ATG7 in PRRSV replication, Marc-145 cells were transfected with control siRNA (NC), simBNIP3, simATG7, or a combination of simBNIP3 and simATG7. At 48 h post-transfection, cells were infected with PRRSV and harvested at 8 h post-infection for Western blotting and RT-qPCR analysis.

**Fig 9:**
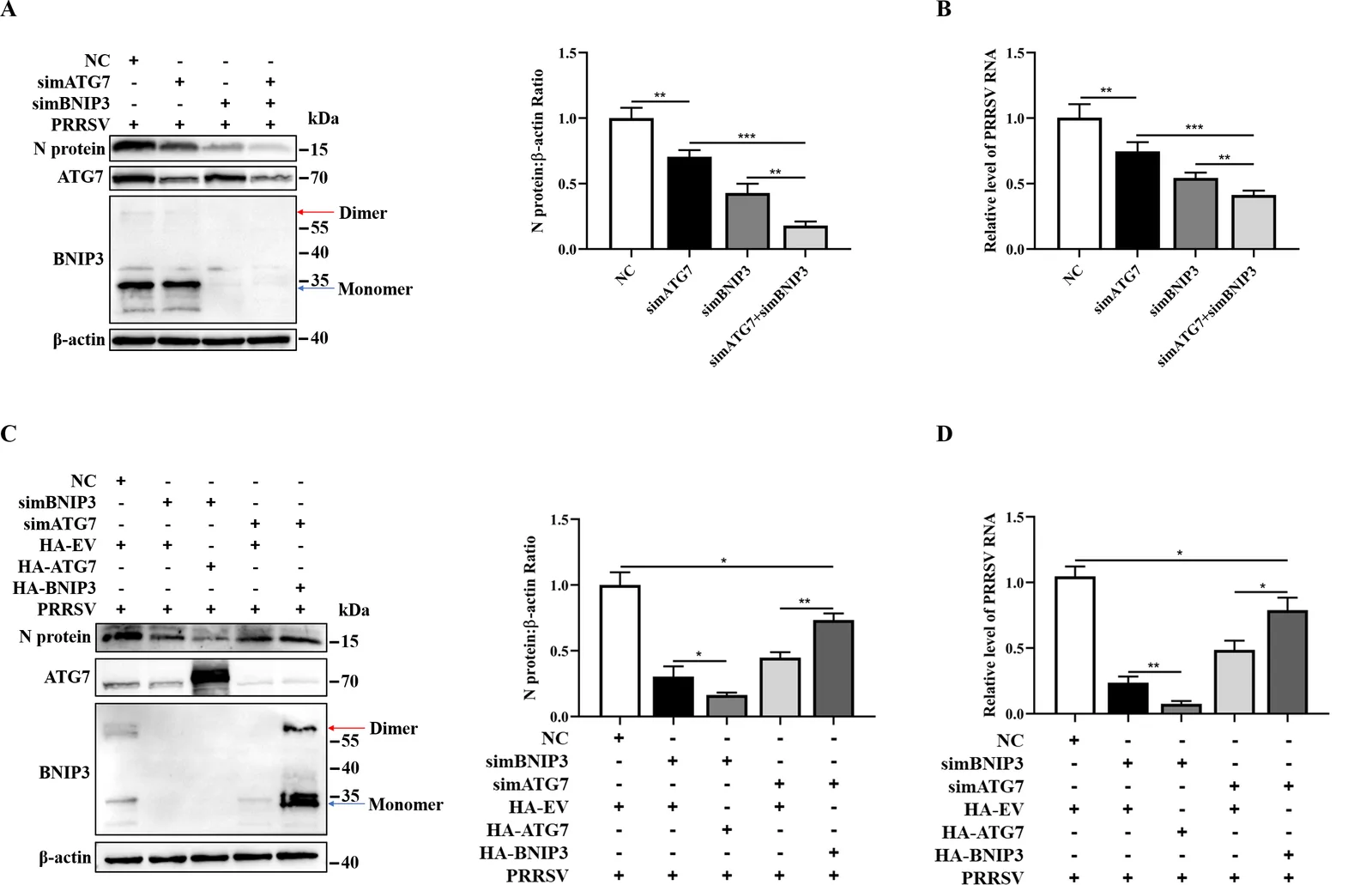
BNIP3 cooperates with ATG7 to regulate PRRSV replication. (**A and B**) Marc-145 cells were transfected with negative control siRNA (NC), simBNIP3, simATG7, or a combination of the two siRNAs for 48 h, prior to PRRSV infection for 8 h. Viral N protein was detected by Western blotting (**A**), with β-actin as the loading control. Viral RNA levels were measured by RT-qPCR and normalized to GAPDH (**B**). (**C and D**) Marc-145 cells were transfected with NC, simBNIP3 or simATG7 for 48 h, followed by a second transfection with HA-empty vector (HA-EV), HA-ATG7, or HA-BNIP3 for 24 h. Cells were then infected with PRRSV for 8 h. Viral N protein was analyzed by Western blotting (**C**). Viral RNA levels were quantified by RT-qPCR and normalized to GAPDH (**D**). Data are presented as mean ± SD. *P < 0.05; **P < 0.01; ***P < 0.001.

Single knockdown of either BNIP3 or ATG7 significantly reduced viral N protein expression and viral RNA levels compared with the NC group. Notably, dual knockdown of BNIP3 and ATG7 resulted in a further reduction in viral N protein and RNA levels compared to either single knockdown, exhibiting a further suppression of PRRSV replication (Fig. 9A and B). These results indicate that BNIP3 and ATG7 function cooperatively, rather than redundantly, to support PRRSV replication.

To further dissect their regulatory relationship, we performed reciprocal knockdown and overexpression rescue experiments. ATG7 overexpression failed to restore viral replication in BNIP3-knockdown cells and instead further decreased viral N protein levels. In contrast, BNIP3 overexpression partially rescued the replication defect induced by ATG7 knockdown although viral production remained significantly lower than that in the NC group (Fig. 9C and D). Collectively, these data suggest that BNIP3 facilitates PRRSV replication partially through an ATG7-independent pathway, likely by providing membrane platforms for replication complex assembly, whereas ATG7 cannot compensate for the loss of BNIP3.

## DISCUSSION

In the present study, we identified BNIP3 as a novel host factor that interacts with PRRSV Nsp9 and mediates exploitation of the autophagic machinery to generate membrane platforms that support viral replication. Mechanistically, Nsp9 is recruited to the mitochondrial outer membrane by binding the transmembrane (TM domain of BNIP3 via its N-terminal 1–449 region. BNIP3-dependent autophagic membranes act as platforms for viral replication complexes, and disruption of the Nsp9–BNIP3 interaction abolishes this process and restricts PRRSV replication. Furthermore, BNIP3 is exploited by PRRSV to block autophagic flux. Collectively, our results establish BNIP3 as a central molecular linker between PRRSV Nsp9 and the host autophagic machinery, which is essential for efficient viral replication.

BNIP3 is a well-characterized mitophagy receptor that localizes to the outer mitochondrial membrane under stress conditions and mediates the clearance of damaged mitochondria through its LIR-dependent interaction with LC3 (17–19). While the canonical function of BNIP3 is to promote autophagic degradation of mitochondria, our study reveals that PRRSV Nsp9 subverts BNIP3 for a non-degradative purpose. Instead of inducing mitophagy, Nsp9 exploits BNIP3 to generate autophagic membranes that support replication complex assembly. This strategy is consistent with the autophagy-subverting mechanism employed by multiple viruses, including hepatitis viruses and coronaviruses, which exploit incomplete autophagy to obtain membrane resources without triggering lysosomal degradation (31–33). Our findings, thus, reveal that a mitophagy receptor can be hijacked to serve this function, extending the functional repertoire of BNIP3 beyond mitophagy and identifying it as a host factor exploited by PRRSV to promote viral replication.

The interaction between Nsp9 and BNIP3 is mediated by discrete structural domains: the TM domain of BNIP3 and the N-terminal 1–449 region of Nsp9. The TM domain of BNIP3 is responsible for its mitochondrial localization (17). Our finding that Nsp9 binds to this domain explains why Nsp9 is recruited to mitochondria in a BNIP3-dependent manner. Notably, the TM domain is also essential for BNIP3 to promote PRRSV replication, suggesting that the mitochondrial membrane localization of the Nsp9-BNIP3 complex is critical for BNIP3’s role in promoting PRRSV replication. The N-terminal 1–449 region of Nsp9 contains the RdRp domain and additional sequences that may mediate protein-protein interactions (12, 34). Future studies will explore whether this region mediates additional interactions with host or viral factors to further orchestrate replication complex assembly. Although the N-terminal 1–449 aa region of Nsp9 was identified as the BNIP3-interacting domain, further fine-mapping is needed to define the minimal binding motif. Such studies may involve generating smaller Nsp9 deletion constructs or employing structural approaches such as co-crystallization or hydrogen-deuterium exchange mass spectrometry.

Notably, overexpression of the TM-deleted BNIP3 mutant (BNIP3 ΔTM) not only failed to support viral replication but also significantly suppressed viral propagation, suggesting a potential dominant-negative effect. This suppression is unlikely to be due to reduced expression of the ΔTM construct, as both WT and ΔTM proteins were expressed at comparable levels (Fig. 3D). Since the TM domain is required for BNIP3 to localize to mitochondria and to interact with Nsp9, the ΔTM mutant cannot bind Nsp9 and, therefore, fails to recruit it to mitochondria. Instead, it might sequester limiting host factors or compete with endogenous BNIP3 for binding to partners such as LC3 or ATG7, thereby disrupting autophagic membrane formation and indirectly suppressing viral replication. Further investigation is needed to distinguish between these possibilities.

One of the key findings of this study is that the LIR motif of BNIP3 is indispensable for its ability to promote PRRSV replication. The LIR motif is required for BNIP3 to bind LC3 and to maintain autophagic flux during infection. Overexpression of a LIR-deficient BNIP3 mutant failed to enhance viral replication, and this mutant retained its interaction with Nsp9 but completely lost colocalization with LC3. These data indicate that the physical interaction between BNIP3 and LC3 is not only essential for autophagy regulation but also for tethering the Nsp9–BNIP3 complex to autophagic membranes. The LIRm mutant data indicate that loss of LC3 binding directly abrogates the formation of the membrane platform required for replication, as the mutant retains Nsp9 interaction but cannot tether the complex to LC3. However, we cannot formally exclude the possibility that the LIR motif may have additional LC3-independent functions, or that other mechanisms may partially compensate for the loss of LC3 binding. It is plausible that BNIP3 acts as a bridging molecule, simultaneously binding Nsp9 via its TM domain and LC3 via its LIR motif, thereby tethering the viral replication complex to the autophagic membrane. This model is supported by our three-color colocalization experiments showing that BNIP3, Nsp9, and LC3 converge at the same subcellular sites.

A notable observation in our study is that BNIP3 knockdown enhanced, rather than suppressed, the BafA1-induced LC3-II accumulation in PRRSV-infected cells. This seemingly paradoxical result can be explained by the impact of PRRSV infection on autophagic flux. PRRSV infection itself blocks autophagosome-lysosome fusion, thereby inhibiting autophagic flux; under these conditions, the LC3-II response to BafA1 is reduced because the virus-imposed blockade suppresses autophagosome turnover, thereby diminishing the number of new autophagosomes that can accumulate during BafA1 treatment. When BNIP3 is knocked down, the virus-imposed blockade is partially relieved, allowing autophagic flux to resume and thereby enabling a stronger LC3-II accumulation upon BafA1 treatment. This de-repression phenotype provides functional evidence that BNIP3 is hijacked by PRRSV to actively block autophagic flux, rather than merely being a passive participant in the process.

To assess whether BNIP3 acts through the canonical autophagy pathway, we selected ATG7 as a key functional partner because ATG7 encodes the E1-like enzyme essential for LC3 lipidation and autophagosome formation, and its knockdown is a well-established strategy to block autophagic flux (35). Knockdown of either BNIP3 or ATG7 reduced viral replication, and double knockdown resulted in a further decrease, indicating that BNIP3 and ATG7 act cooperatively in promoting PRRSV replication. Notably, overexpression of ATG7 failed to rescue the replication defect caused by BNIP3 knockdown and instead further suppressed viral replication, suggesting that BNIP3 contributes to viral replication not only through ATG7-dependent autophagic flux but also through additional mechanisms. One such mechanism may be that BNIP3-associated membranes serve as membrane platforms for replication complex assembly independent of ATG7.

This interpretation is further supported by rapamycin treatment experiments. Despite inducing robust LC3-II accumulation, rapamycin failed to promote PRRSV replication and instead reduced viral titers. This can be explained by two mechanisms: first, rapamycin downregulates BNIP3 expression, reducing the availability of membrane platforms; second, excessive autophagy activation may promote autophagosome maturation and degradation, eliminating the membrane platforms that the virus relies on. These findings support the model that BNIP3-mediated membrane platform formation, rather than autophagosome accumulation alone, is the critical determinant of PRRSV replication efficiency. Of note, it has been reported that rapamycin downregulates PRRSV protein synthesis but enhances viral replication (36, 37); the net effect on viral titers likely reflects the balance between this pro-replication effect and the opposing effect of BNIP3 downregulation.

Several questions remain to be addressed. First, it is unclear whether BNIP3 is specifically recruited to replication sites by Nsp9 or whether Nsp9 passively follows BNIP3 to mitochondria. Our data show that Nsp9 binds to the TM domain of BNIP3, but whether other viral or host proteins contribute to this process remains to be determined. Second, the source of autophagic membranes used as platforms, whether they originate from mitochondria, endoplasmic reticulum, or other organelles, requires further characterization. The use of specific organelle markers and correlative light and electron microscopy may help resolve this issue. Finally, although our work focused on Marc-145 cells and primary PAMs, it will be important to validate the functional relevance of the Nsp9-BNIP3 axis *in vivo*, for example, using cell-type-specific BNIP3 knockout pigs or pharmacological inhibitors targeting the Nsp9-BNIP3 interface.

**Fig 10:**
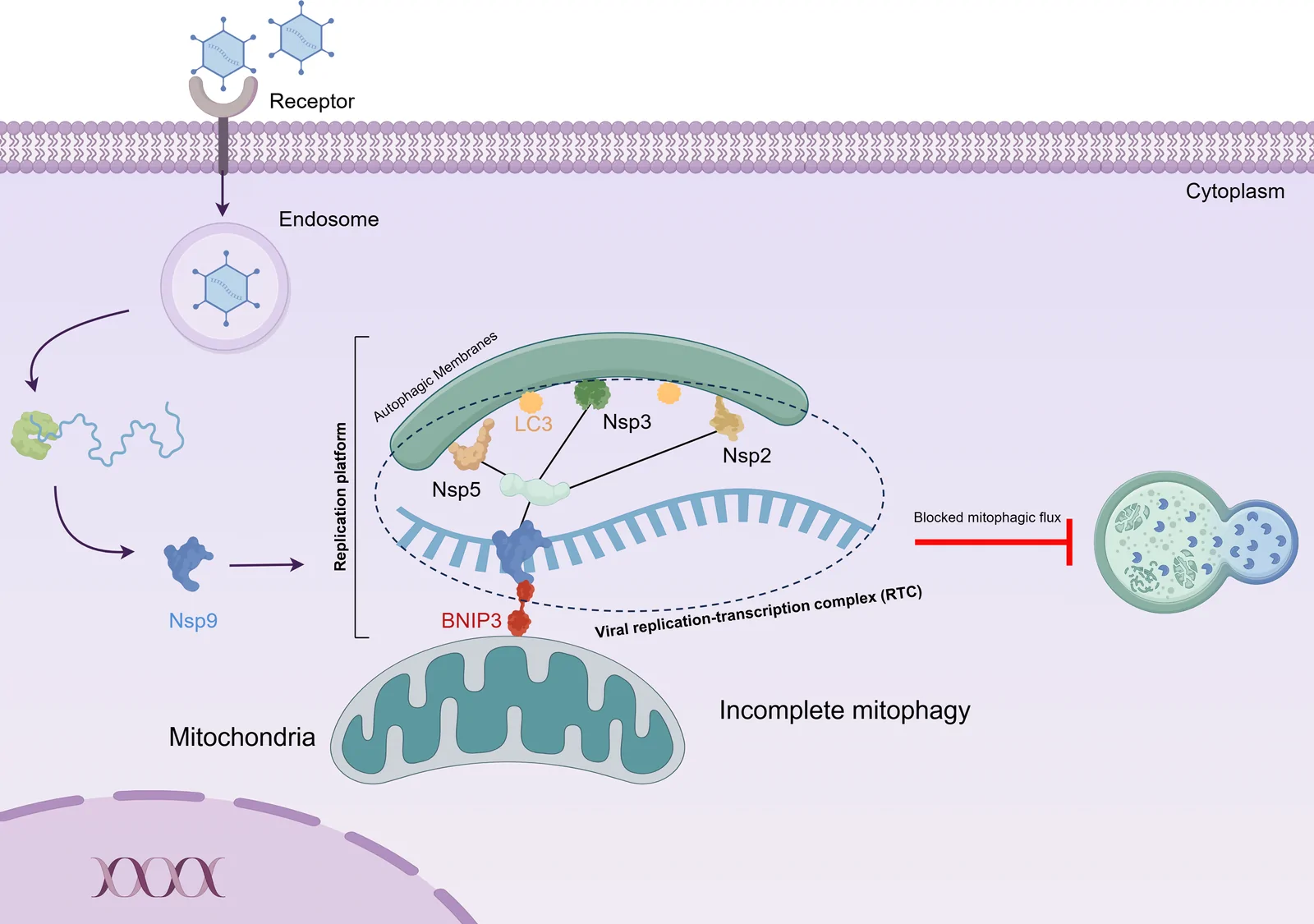
Schematic model of PRRSV Nsp9 hijacking BNIP3 to block autophagic flux and utilize autophagic membranes as replication platforms. Nsp9 interacts with the transmembrane (TM) domain of BNIP3 through its N-terminal region, recruiting viral replication complexes to mitochondrial membranes. Nsp9 blocks autophagic flux, resulting in incomplete mitophagy. BNIP3-associated autophagic membranes serve as platforms for replication complex assembly. BNIP3 engages LC3 through its LIR motif, and this LIR-dependent interaction with LC3 is essential for BNIP3 to promote viral replication.

Based on our findings, we propose a working model in which PRRSV Nsp9 hijacks BNIP3-mediated autophagy to provide membrane platforms for viral replication (Fig. 10). Nsp9 binds to the TM domain of BNIP3, and BNIP3 engages LC3 through its LIR motif, thereby tethering replication complexes to autophagic membranes. Disruption of this axis severely impairs viral replication. Together, our findings identify BNIP3 as a critical host factor exploited by PRRSV to promote viral replication and highlight its potential as a target for antiviral intervention.

## MATERIALS AND METHODS

### Cells and viruses

Marc-145 cells were cultured in Dulbecco’s modified Eagle’s medium (DMEM) supplemented with 10% fetal bovine serum (FBS) at 37°C with 5% CO₂. Primary porcine alveolar macrophages (PAMs) were isolated from 4-week-old piglets via lung lavage using PBS containing 0.5% EDTA, as described previously (38). After isolation, cells were centrifuged at 500 × *g*, resuspended in RPMI-1640 medium supplemented with 10% FBS, and seeded into 6-well plates. Non-adherent cells were discarded after 2 h of incubation. PRRSV strain XHGD was propagated and titrated in Marc-145 cells, and viral titers were determined using the 50% tissue culture infectious dose (TCID₅₀) assay. Unless otherwise indicated, all PRRSV infections were performed with the XHGD strain. The heterologous NADC30-like strain was used for cross-strain validation experiments and was also preserved in our laboratory. For virus titration in infection supernatants, Marc-145 cells were seeded into 96-well plates and inoculated with 10-fold serial dilutions of the harvested culture supernatants. After 72 h of incubation, cytopathic effects (CPE) were recorded, and TCID_50_ titers were calculated using the Reed-Muench method.

### Antibodies and chemicals

Mouse anti-PRRSV N protein (JN0401) was obtained from Beijing Jinnuo Baitai Biotechnology. Alpaca anti-PRRSV Nsp9 Nanobody (Nb8) (RVV29001) was purchased from AntibodySystem. Rabbit anti-HA tag (3724) was purchased from Cell Signaling Technology. Mouse anti-BNIP3 (68091-1-Ig), rabbit anti-GFP (50430-2-AP), rabbit anti-β-Tubulin (10094-1-AP), and mouse anti-β-actin (66009-1-Ig) were purchased from Proteintech. Rabbit anti-BNIP3 (R23308) was purchased from Zen-BioScience. Mouse anti-Flag (AF519), mouse anti-HA (AH158), mouse IgG (A7028), rabbit IgG (A7016), Protein A + G agarose (P2055), Mito-Tracker Red CMXRos (C1035), Lipo8000 transfection reagent (C0533), cell lysis buffer (P0013), and BCA protein assay kit (P0010) were purchased from Beyotime. Rfect siRNA/miRNA transfection reagent (11013) was purchased from Baidai Biotechnology. Rabbit anti-Flag (T20008) was purchased from Abmart. Alexa Fluor 594 goat anti-rabbit IgG (A11012), Alexa Fluor 488 goat anti-mouse IgG (A11011), Alexa Fluor 647 goat anti-mouse IgG (A21235), Alexa Fluor 647 goat anti-rabbit IgG (A21244), Goat anti-Mouse IgG (H + L) Secondary Antibody HRP (31430), and Goat anti-Rabbit IgG (H + L) Secondary Antibody HRP (31460) were purchased from Thermo Fisher Scientific. Goat anti-Alpaca IgG (H + L) antibody (HRP) (053-404-005) was purchased from AlpVHHs. Rabbit anti-LC3B (ab192890) and donkey anti-mouse IgG H&L (Alexa Fluor 405) (ab175658) were obtained from Abcam. Mouse anti-dsRNA (J2 clone, 10010500) was purchased from SCICONS. Bafilomycin A1 (BafA1, HY-100558) and Rapamycin (HY-10219) were purchased from MedChemExpress. Enhanced chemiluminescence (ECL, MA0186-2) was purchased from Meilunbio. ClonExpress Ultra One Step Cloning Kit (C115), HiScript III All-in-one RT SuperMix (R333-C1), and ChamQ Universal SYBR qPCR Master Mix (Q711) were purchased from Vazyme. TRIzol universal reagent (DP424) was obtained from TIANGEN.

### Plasmid construction and mutagenesis

cDNAs encoding PRRSV proteins (Nsp1α, Nsp1β, Nsp2, Nsp3, Nsp4, Nsp5, Nsp7, Nsp9, Nsp10, Nsp11, Nsp12, GP2a, GP3, GP4, GP5, M, N) were cloned into Flag-tagged vectors pCAGGS using ClonExpress Ultra One Step Cloning Kit. Full-length porcine BNIP3 and full-length porcine ATG7 were individually cloned into the HA-tagged pCAGGS vector to generate two separate plasmids. A series of BNIP3 truncation mutants (1–109 aa, 110–196 aa, 1–163 aa, 119–196 aa, 164–183 aa, 184–196 aa) and Nsp9 (1–177 aa, 178–449 aa, 450–643 aa, 1–449 aa, 178–643 aa) were generated by PCR and verified by sequencing. The LIR motif mutant of BNIP3 (LIRm, W18A/L21A) was generated by gene synthesis. The mitophagy reporter Mito-GFP-mCherry, autophagic flux reporters LC3-GFP-mCherry, and LC3-GFP plasmids were preserved in our laboratory.

### Transfection and RNA interference

Marc-145 cells were transfected with plasmids using Lipo8000 transfection reagent according to the manufacturer’s instructions. For siRNA-mediated knockdown, cells were transfected with siRNAs at a final concentration of 20 nM using Rfect siRNA/miRNA transfection reagent. All siRNA sequences used in this study are listed in Table 1. A non-targeting siRNA was used as the negative control (NC). The simATG7 sequence was derived from a previous study (39) and validated for knockdown efficiency in Marc-145 cells under the same conditions as BNIP3 (48 h, 20 nM), as shown in Fig. 9A. Knockdown efficiency was confirmed by Western blotting at 48 h post-transfection.

**TABLE 1:** siRNAs used in this study

siRNA name	Species	Sequence (5'→3')
simBNIP3-1	Monkey	(Sense) CGUUCCAGCCUCGGUUUCUAUUUAU
		(Antisense) AUAAAUAGAAACCGAGGCUGGAACG
simBNIP3-2	Monkey	(Sense) GAGGAACACGAGCGUCAUGAA
		(Antisense) UUCAUGACGCUCGUGUUCCUC
sisBNIP3-1	Sus scrofa	(Sense) CGUUCCAGACUCCGUUUCUAUUUAU
		(Antisense) AUAAAUAGAAACGGAGUCTGGAACG
sisBNIP3-2	Sus scrofa	(Sense) GAGGAACACGAGCGUGAUGAA
		(Antisense) UUCAUCACGCUCGUGUUCCUC
simATG7	Monkey	(Sense) GCCCACAGAUGGAGUAGCA
		(Antisense) UGCUACUCCAUCUGUGGGC

### PRRSV infection and sample collection

Marc-145 cells were inoculated with PRRSV at an MOI of 10 unless otherwise stated. After 1 h adsorption at 37°C, unbound virus was removed by washing with PBS, and cells were cultured in maintenance medium (DMEM with 2% FBS). At indicated time points, cells were harvested for Western blotting or RNA extraction.

### Western blotting

Cells were lysed in lysis buffer supplemented with protease and phosphatase inhibitors. Protein concentrations were determined by BCA protein assay kit. Equal amounts of protein were separated by SDS-PAGE and transferred onto nitrocellulose membranes. Membranes were blocked with 5% non-fat milk and incubated with indicated antibodies at 4°C overnight. After incubation with HRP-conjugated secondary antibodies for 1 h at room temperature, protein signals were detected using ECL with a Tanon5200 imaging system (Tanon Science & Technology, Shanghai, China). Band intensities were quantified using ImageJ 1.8.0.

### Reverse-transcription quantitative PCR

Total RNA was extracted using TRIzol universal reagent and reverse-transcribed using HiScript III All-in-one RT SuperMix. qPCR was performed using ChamQ Universal SYBR qPCR Master Mix on QuantStudio 1 Plus (Thermo Fisher Scientific). The primers for PRRSV ORF7 and sus scrofa GAPDH were used as previously described (40) and are listed in Table 2. Relative viral RNA levels were normalized to cellular GAPDH mRNA and calculated using the 2^⁻ΔΔCt^ method.

**TABLE 2:** Primers for RT-qPCR used in this study

Target gene	Species	Sequence (5'→3')
ORF7	PRRSV	(Sense) AAACCAGTCCAGAGGCAAGG
		(Antisense) GCAAACTAAACTCCACAGTGTAA
GAPDH	Monkey	(Sense) GAAGGTGAAGGTCGGAGTCA
		(Antisense) CATGTAAACCATGTAGTTGAGGTC
GAPDH	Sus scrofa	(Sense) CCTTCCGTGTCCCTACTGCCAAC
		(Antisense) GACGCCTGCTTCACCACCTTCT

### Confocal microscopy and colocalization analysis

Marc-145 cells grown on coverslips were transfected as indicated. At 24 h post-transfection, cells were fixed with 4% paraformaldehyde, permeabilized with 0.1% Triton X-100, and blocked with 5% bovine serum albumin. Cells were incubated with appropriate primary antibodies, followed by Alexa Fluor 405, 488-, 594-, or 647-conjugated secondary antibodies. Nuclei were counterstained with DAPI. Images were acquired using a Leica STELLARIS 8 FALCON confocal microscope with a 63 × oil-immersion objective. Colocalization analysis was performed via the Coloc 2 plugin in ImageJ 1.8.0 to calculate Pearson’s correlation coefficient (PCC, Robs). Robs > 0.50 was considered significant colocalization, as previously described (41). Fluorescence intensity profiles were plotted to verify spatial overlap of fluorescent signals.

For puncta quantification, images were processed using ImageJ 1.8.0. Individual cells were outlined manually as regions of interest. Yellow puncta (GFP^+^mCherry^+^, representing autophagosomes) and red-only puncta (GFP⁻mCherry^+^, representing autolysosomes) were counted using the “Find Maxima” function after threshold adjustment. At least 20 cells per condition from three independent experiments were analyzed.

### Co-immunoprecipitation

Cells were lysed in lysis buffer. A portion of the whole-cell lysate was reserved as the input sample. The remaining lysate was divided equally and incubated with specific primary antibodies or control IgG at 4°C overnight. Protein A + G agarose beads were then added for an additional 6 h of incubation. The beads were then washed three times with wash buffer (50 mM Tris-HCl [pH 7.4], 200 mM NaCl, 2 mM EDTA, 1% Triton X-100, 0.01% SDS). Bound proteins were eluted and analyzed by Western blotting.

### Immunoelectron microscopy

Marc-145 cells co-expressing Flag-Nsp9 and HA-BNIP3 were fixed with 4% paraformaldehyde and 0.1% glutaraldehyde, embedded in Lowicryl K4M, and ultrathin-sectioned. Sections were incubated with mouse anti-Flag and rabbit anti-HA primary antibodies, followed by 4 nm gold-conjugated anti-mouse and 12 nm gold-conjugated anti-rabbit secondary antibodies. Samples were observed using a HT7800 transmission electron microscope (Hitachi, Tokyo, Japan).

### Autophagic flux assay

Autophagic and mitophagic flux were evaluated using LC3-GFP-mCherry and Mito-GFP-mCherry reporter systems, respectively. Yellow puncta (GFP^+^mCherry^+^) represent unclosed autophagosomes/mitophagosomes, whereas red-only puncta (GFP^-^mCherry^+^) represent mature autolysosomes/mitolysosomes (42). For BafA1 treatment, cells were incubated with 100 nM BafA1 for 4 h before collection. For rapamycin treatment, cells were treated with 100 μM rapamycin for 3 h prior to infection and maintained in the presence of rapamycin throughout the infection period (10 h). Cellular LC3-II levels were detected by Western blotting, and LC3-II/β-actin ratios were quantified using ImageJ 1.8.0.

### Statistical analysis

All experiments were performed at least three independent times. Data are presented as mean ± standard deviation. Statistical significance was determined by Student’s *t*-test using GraphPad Prism 8. Significance levels are indicated as follows: *P < 0.05; **P < 0.01; ***P < 0.001; ****P < 0.0001; ns (not significant), *P* > 0.05. Pearson’s correlation coefficients were calculated using the Coloc 2 plugin in ImageJ 1.8.0.

## Data Availability

All data generated or analyzed during this study are presented in the article and the supplemental material.

## SUPPLEMENTAL MATERIAL

Supplemental material jvi.01314-26-s0001.pdf Figures S1 to S4.

ASM does not own the copyrights to Supplemental Material that may be linked to, or accessed through, an article. The authors have granted ASM a non-exclusive, world-wide license to publish the Supplemental Material files. Please contact the corresponding author directly for reuse.
