# Supplementary material for: PRRSV Nsp9 hijacks BNIP3-mediated autophagy to provide membrane platforms for viral replication complexes
Source: J Virol. 2026 Sep 24;100(10):e01314-26. doi: 10.1128/jvi.01314-26 (PMC13712842; doi:10.1128/jvi.01314-26)
Supplement: Supplementary file 1 [file jvi.01314-26-s0001.pdf]

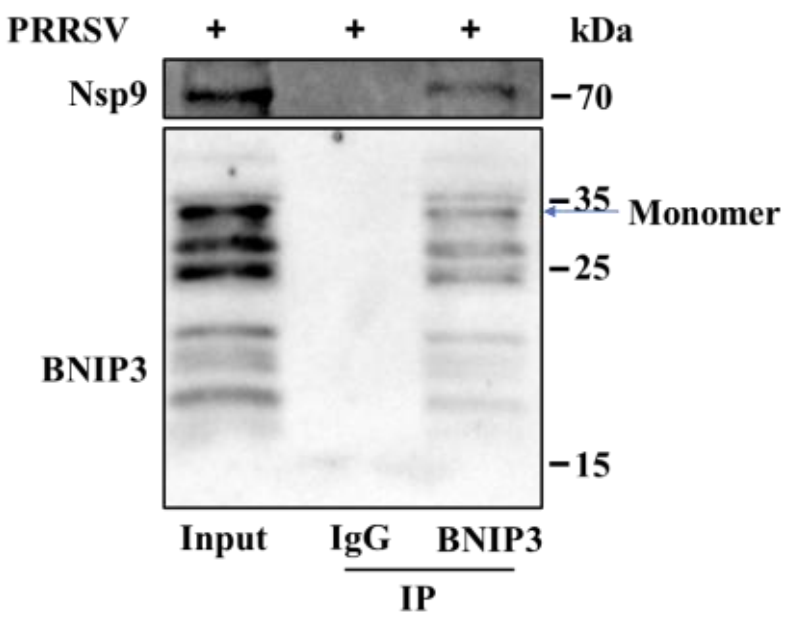

FIG S1 Endogenous co-immunoprecipitation (Co-IP) of BNIP3 and Nsp9 in NADC30-like-infected Marc-145 cells. Following 36 h of infection with NADC30-like, cell lysates were subjected to immunoprecipitation (IP) with mouse anti-BNIP3 antibody or control IgG, and the precipitates were analyzed by immunoblotting (IB) with alpaca anti-Nsp9 and rabbit anti-BNIP3 antibodies. Input samples served as a loading control.

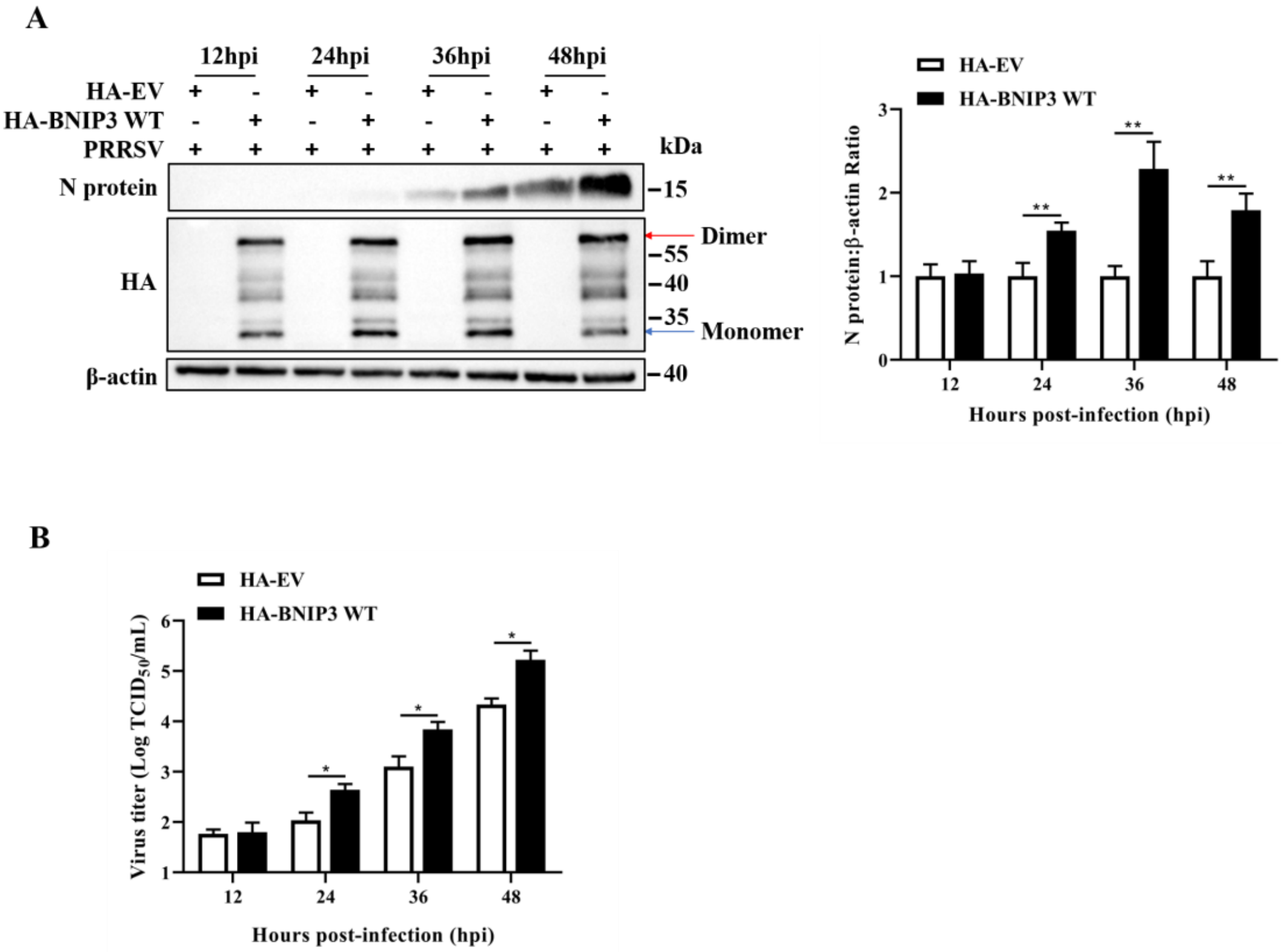

FIG S2 Overexpression of BNIP3 promotes NADC30-like replication. Marc-145 cells were transfected with HA-empty vector (HA-EV) or HA-BNIP3 for 24 h, then infected with NADC30-like at an MOI of 1 for 12, 24, 36, and 48 h. (A) Viral N protein expression was analyzed by Western blotting. HA tag was detected to confirm

BNIP3 overexpression. β-actin served as loading control. (B) Viral titers in culture supernatants were determined by $TCID_{50}$ assay. Data are presented as mean ± SD. *P < 0.05; **P < 0.01.

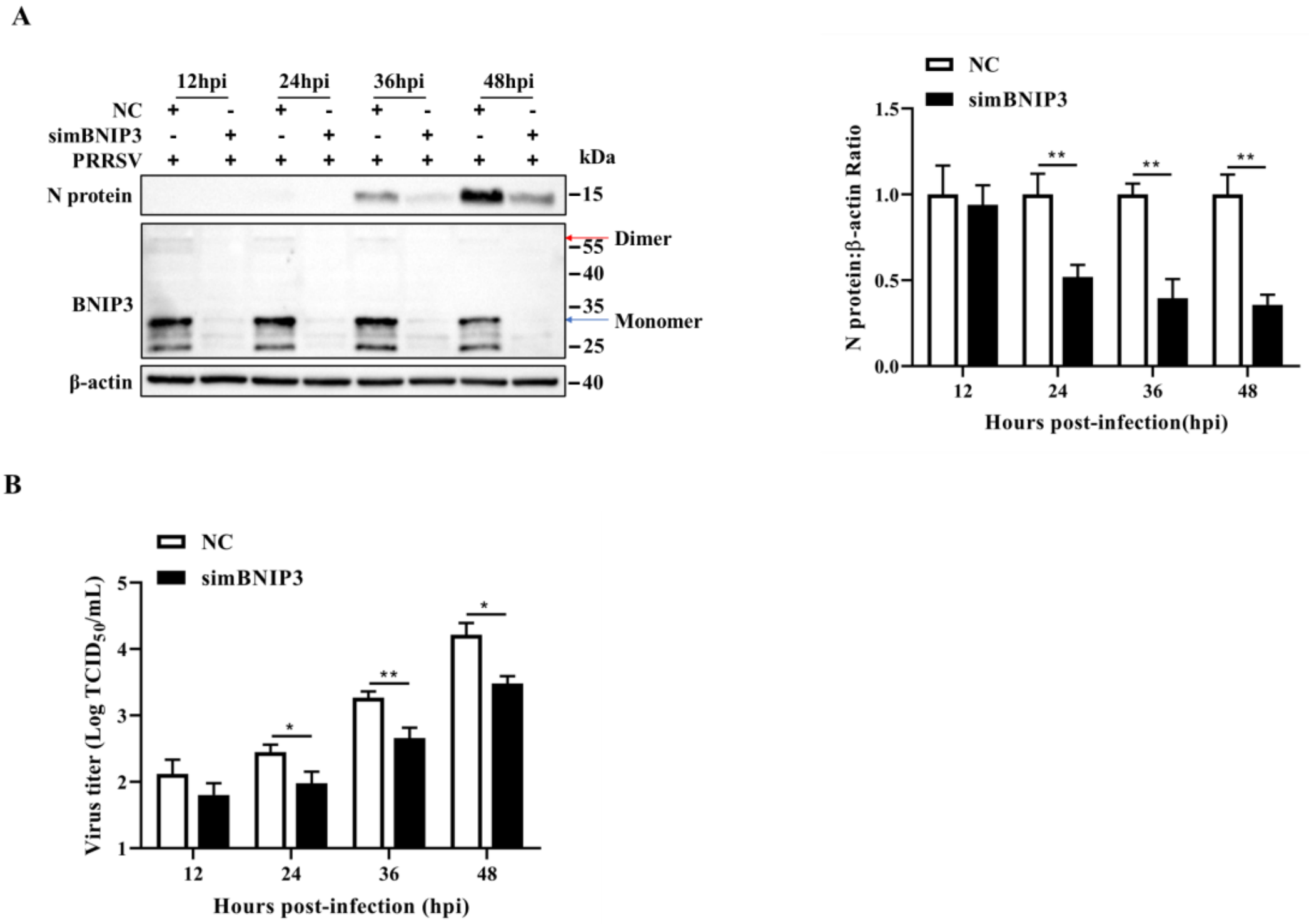

FIG S3 Knockdown of BNIP3 suppresses NADC30-like replication. Marc-145 cells were transfected with control siRNA (NC) or simBNIP3 for 48 h, then infected with NADC30-like at an MOI of 1 for 12, 24, 36, and 48 h. (A) Viral N protein expression was analyzed by Western blotting. BNIP3 levels were detected to confirm knockdown efficiency. β-actin served as loading control. (B) Viral titers in culture supernatants were determined by $TCID_{50}$ assay. Data are presented as mean ± SD. *P < 0.05; **P < 0.01.

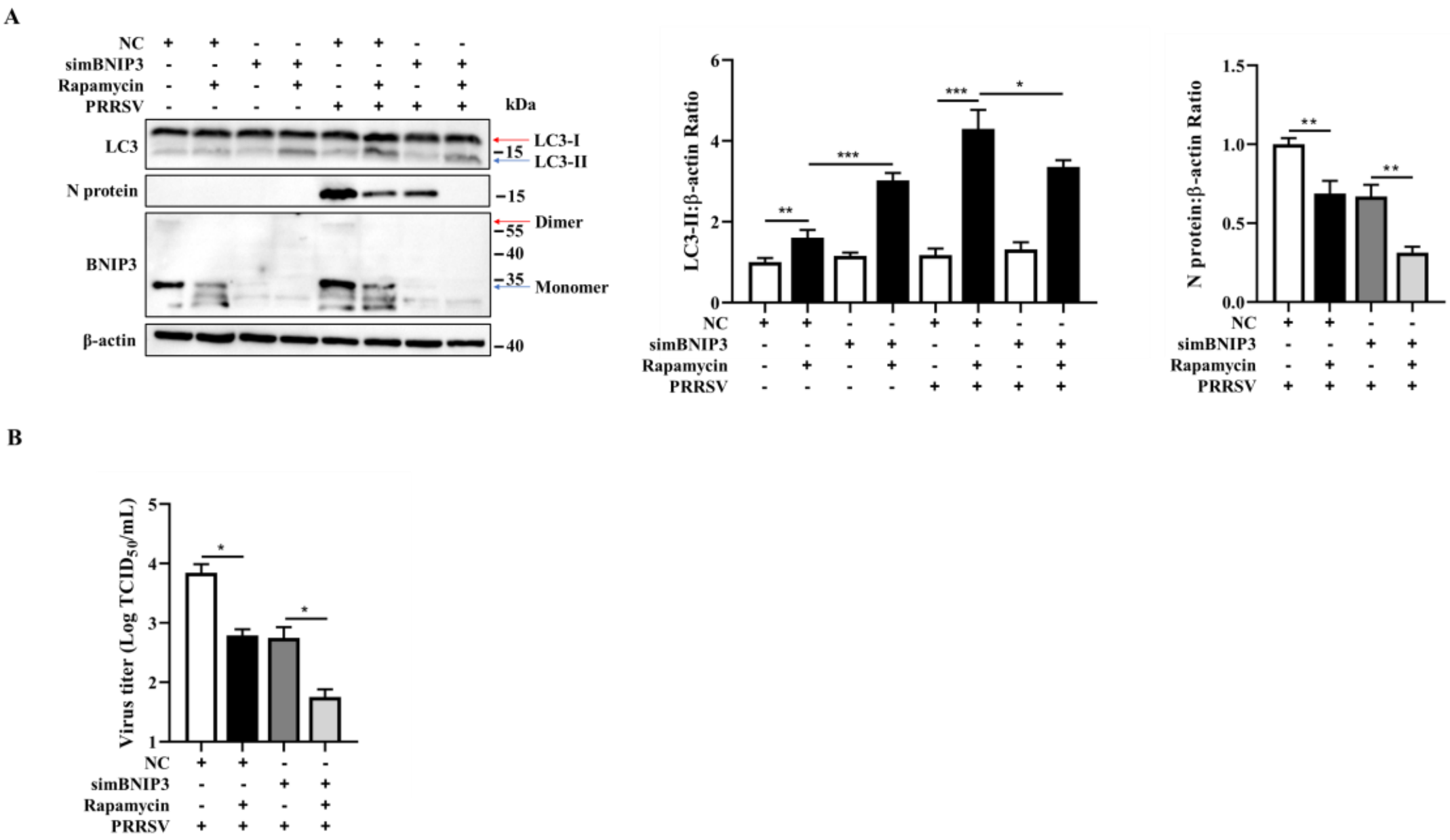

FIG S4 Rapamycin treatment fails to rescue PRRSV replication in BNIP3-knockdown cells. (A) Marc-145 cells were transfected with control siRNA (NC) or siBNIP3 for 48 h, then treated with DMSO or rapamycin (100 μM) for 3 h prior to mock infection or PRRSV infection. Cells were harvested at 10 hpi. LC3-II, BNIP3, and N protein expression were analyzed by Western blotting. β-actin served as loading control. (B) Viral titers in culture supernatants from cells treated as in (A) were determined by $TCID_{50}$ assay. Data are presented as mean ± SD. *$P < 0.05$; **$P < 0.01$; ***$P < 0.001$.
